# Analysis of drug-drug interactions between psychiatric drugs in spontaneous adverse drug reaction reports from EudraVigilance

**DOI:** 10.1007/s00210-025-04956-5

**Published:** 2026-01-22

**Authors:** Diana Dubrall, Patrick Christ, Miriam Böhme, Martina Hahn, Matthias Schmid, Catharina Scholl

**Affiliations:** 1 Institute for Medical Biometry, Informatics and Epidemiology, University Hospital Bonn, Venusberg-Campus 1, 53127 Bonn, Germany; 2 Research Division, Federal Institute for Drugs and Medical Devices (BfArM), Kurt-Georg-Kiesinger-Allee 3, 53175 Bonn, Germany; 3 Department of Mental Health, Varisano Hospital Frankfurt Hoechst, Gotenstr. 6-8, 65929 Frankfurt, Germany; 4 Department of Psychiatry, Psychosomatics and Psychotherapy, University Hospital Frankfurt, Heinrich-Hoffmann-Str. 10, 60528 Frankfurt, Germany; 5 Department of Pharmacology and Clinical Pharmacy, Philipps-University Marburg, Karl-Von-Frisch-Strasse 2, 35043 Marburg, Germany

**Keywords:** Drug-drug interactions, Spontaneous reports, Adverse drug reaction reports, Psychiatric drugs, Spontaneous reporting systems, Psychopharmacoepidemiology

## Abstract

In psychiatric care, polypharmacy is common practice. The more drugs are used, the higher the likelihood of drug-drug interactions (DDI) and, consequently, the occurrence of adverse drug reactions (ADR). The aim of our study was to identify DDI between psychiatric drugs and to analyze their associated factors in spontaneous ADR reports contained in the European ADR database EudraVigilance. Out of 9287 ADR reports from EudraVigilance, 1811 reports with potential DDI (pDDI) were detected by the ABDATA interaction analysis. During the individual case assessment of the most frequently identified pDDI (*n* = 1342), 206 reports with DDI were found. These 206 reports were analyzed descriptively. Patients who experienced DDI were on average 52.6 (± 18.8) years old. Reports describing DDI were clearly more often classified as serious than those without DDI (81.1% versus 59.0%, *p*-value < 0.005). The DDI most frequently identified were *tachycardia and QT prolongation* (23.3%, 48/206), *extrapyramidal symptoms* (16.0%, 33/206), *sedation* (11.7%, 24/206) and *seizures* (11.7%, 24/206). Mirtazapine–quetiapine (26.3%, 10/48) was the interacting drug pair most commonly associated with *tachycardia and QT prolongation*. Patients experiencing *tachycardia and QT prolongation* were, on average, older (63.0 (± 16.2) years) and took more drugs (8.0 (± 5.3)) than the overall group of patients with DDI (6.0 (± 3.9)). DDI pose a considerable risk to patients treated with several combinations of psychiatric drugs. Checks for interactions and tight monitoring after initiating drug therapy can help to avoid serious DDI. Special attention should be paid to older patients taking more than one QT-interval prolonging drug.

**Supplementary Information:**

The online version contains supplementary material available at 10.1007/s00210-025-04956-5.

## Introduction

In Germany the prevalence of diagnoses of mental health disorders increased previously (Thom et al. 2024). Likewise, an upward trend was observed for the number of drug prescriptions of antidepressants and antipsychotics (Seifert et al. 2025).

In psychiatric care, it is common practice that patients with severe mental illness often receive combinations of multiple antidepressants, antipsychotics, mood stabilizers, anxiolytics, hypnotics, antihistamines, and anticholinergics (Baandrup 2020; Hu et al. 2022; Stassen et al. 2022) resulting in an increased risk to develop drug-drug interactions (DDI) (Wolff et al. 2021). Thereby, the likelihood of DDI increases the more drugs are used. Although combination therapy might be superior to monotherapy in some cases (Azorin and Simon 2020; Correll et al. 2009; Möller et al. 2014; Tiihonen et al. 2019), potential DDI (pDDI) should be taken seriously as these could cause harmful adverse drug reactions (ADR) (Azorin and Simon 2020; Kennedy et al. 2013; Kratz and Diefenbacher 2019; Seifert et al. 2025), affect the efficacy of drug therapy (Azorin and Simon 2020) and thus may lead to treatment non-compliance of the patient (Chaudhari et al. 2017; Velligan et al. 2009). In the case of ADRs, these may initiate drug prescribing cascades, further increasing the likelihood of DDI (Doherty et al. 2022). Moreover, common ADRs to psychiatric drugs such as weight gain or cardiovascular ADRs may additionally increase the risk to develop somatic comorbidities (De Hert et al. 2009; Virk et al. 2004).

In clinical practice, some of the DDI can be avoided or detected at an early stage by appropriate measures like prescribing drugs with a lower potential of DDI, choosing suitable dose adjustments and performing therapeutic drug monitoring (TDM) (Kratz and Diefenbach 2019, Tannenbaum and Sheehan 2014).

One appropriate tool for analyzing ADRs and DDIs occurring in daily clinical practice is the spontaneous reporting system, as demonstrated in other studies (Hult et al. 2020; Jiang et al. 2022; Leone et al. 2010; Letinier et al. 2020; Magro et al. 2020; Mirosevic Skvrce et al. 2011) and in one of our previous analyses (Dubrall et al. 2025). In this analysis, we were able to identify known DDI between drugs used as antidepressants, antipsychotics and mood stabilizers (hereafter referred to as psychiatric drugs) and somatic medications.

The analysis presented in this manuscript describes the identified DDI between two psychiatric drugs.

The aim of this study was to determine the number of ADR reports from Germany in EudraVigilance, in which *already known* DDI between two psychiatric drugs were reported. Additionally, we investigated the most commonly identified DDI and their associated factors.

## Material and methods

### Definitions

ADRs (definition is described in literature) can be reported by Health Care Professionals (HCP, e.g., physicians, pharmacists) who are obliged by their professional code of conduct to report ADRs or non-Health Care Professionals (non-HCP, e.g., consumers) (GVP Module VI 2017).

DDI can either be pharmacodynamic or pharmacokinetic (Hiemke et al. 2018, Zeitlinger 2016). A pharmacodynamic DDI occurs when two drugs affect the activity of each other and, among others, lead to an increased or decreased pharmacological effect of one of these drugs, or to an ADR, which would not manifest, or would manifest to a lesser extent, with the individual drugs alone. In pharmacokinetic DDI the absorption, distribution, metabolism or excretion of one of the drugs is altered. The most prominently involved enzymatic system in pharmacokinetic DDI related to psychiatric drugs is the cytochrome P450 system. Pharmacokinetic DDI can either lead to increased (potentially) toxic serum concentrations or decreased serum concentrations below the therapeutic level.

### EudraVigilance

EudraVigilance is the ADR database of the European Medicines Agency. In EudraVigilance, all spontaneous ADR reports received from one of the member states of the European Economic Area are included (Human regulatory n.d.). ADRs are coded in accordance with MedDRA terminology (MedDRA Dictionary n.d.) and drugs with the EudraVigilance medicinal product dictionary (Extended EudraVigilance medicinal product dictionary n.d.). MedDRA terminology (MedDRA Dictionary n.d.) consists of five different hierarchical levels allowing aggregated and more detailed analyses. The preferred term (PT) level of MedDRA terminology describes, among other things, symptoms, diagnoses, investigations and laboratory results. These PTs are grouped based upon anatomy, pathology, physiology, etiology or function on the high level term (HLT) level, and then on the high level group term (HLGT) level. The highest level of analyses is the system organ class (SOC) level describing the organ system in which the ADR occurs.

At the submission of each ADR report, the reporter classifies which drug/s is/are assumed to be suspected, interacting or concomitant (GVP Module VI 2017).

#### Extraction of ADR reports from EudraVigilance

All spontaneous ADR reports from Germany received between 01/2017 and 12/2021 reported for adults (> 17 years) in which antidepressants (anatomical therapeutic classification (ATC) (ATC code n.d.) N06A), antipsychotics (ATC N05A) and mood stabilizers (e.g., carbamazepine) were reported as suspected/interacting (*n* = 9665) were extracted (the complete list of drugs analyzed is presented in Online Resource 1). ADR reports describing intentional overdoses and suicide attempts were excluded leading to a total dataset of *n* = 9276 reports. Within these 9276 reports, all drugs reported as suspected/interacting or concomitant were grouped as pairs, and every combination was evaluated for pDDI.

**Fig. 1:**
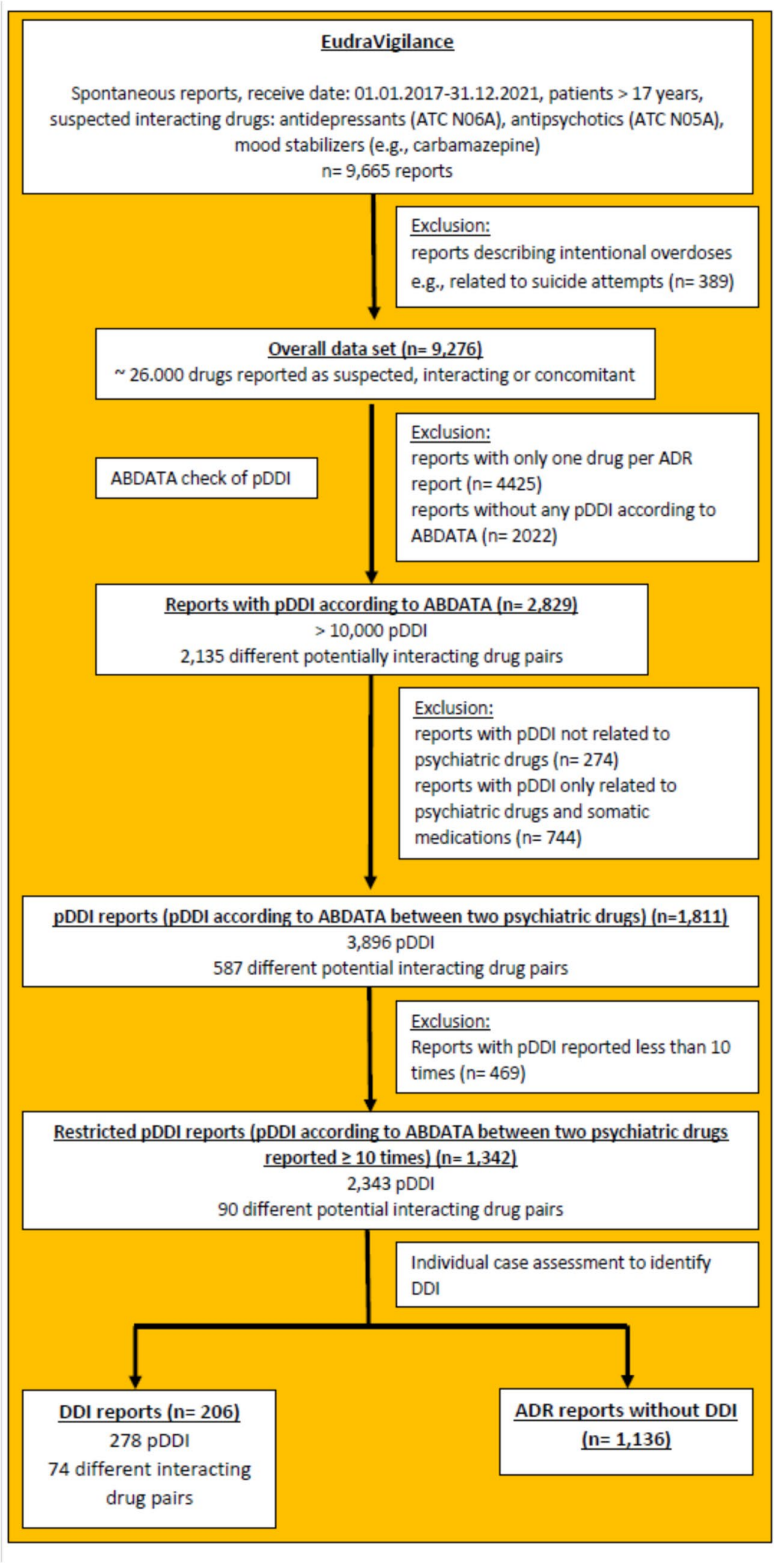
Identification of ADR reports and pDDI related to psychiatric drugs and their analyses.

Figure 1 shows the extraction of the ADR reports from EudraVigilance, the identification of the pDDI by using the ABDATA interaction check and the identified DDI during the individual case assessment.

### ABDATA interaction analysis

The ABDADatenbank (ABDATA Pharma Data Service (a division of Avoxa – Mediengruppe Deutscher Apotheker GmbH), data status as of January 1, 2022) was used to detect pDDI (ABDATA Pharma-Daten-Service 2025). The ABDATA database consists of 5 modules. For our analysis, we used the information (i) on active ingredients from the medicinal product module and (ii) the drug therapy safety (AMTS, Arzneimitteltherapiesicherheit) module was used for specific interaction analysis. The interaction database of the ABDADatenbank is based on documented sources such as summaries of product characteristics, scientific literature, and relevant books, which are precisely  recorded for each DDI. Based on that, each DDI of the AMTS module is classified according to its clinical relevance, source of evaluation, data availability, and plausibility of mechanism. An evidence rating is assigned to each DDI ranging from “well documented” to “insufficient information”. This means that well-established clinical studies indicate high data quality, while contradictory or very sparse case reports are considered insufficient. Further information for each DDI includes the pharmacological effect, the mechanism that leads to the DDI, possible ADRs, and recommended actions. In addition, each DDI is given a clinical relevance rating—from “contraindicated” to “serious” to “no relevant interaction expected.” Only clinically relevant DDI were included in our analysis.

#### Identification of reports with pDDI according to ABDATA

Overall, around 26,000 drugs were reported as suspected/interacting or concomitant in the 9276 reports (Fig. 1). At first, all ADR reports with only one reported drug (*n* = 4425) and those without a pDDI according to ABDATA (*n* = 2022) were excluded leading to 2829 reports. Another 274 reports and 744 reports were omitted because the identified pDDI was either not associated with a psychiatric drug or described a pDDI solely between a psychiatric drug and somatic medications. In the end, 1811 reports with 587 different potentially interacting psychiatric drug pairs and 3896 pDDI were identified (list of potentially interacting drug pairs in Online Resource 2) (hereafter, referred to as pDDI reports). For our individual case assessment, we restricted our dataset to potentially interacting drug pairs reported more than 10 times (in the following: restricted pDDI reports). Finally, 1342 reports with 2343 pDDI and 90 different potential interacting psychiatric drug pairs remained. Notably, these restricted pDDI reports contain potential DDI identified by the ABDATA interaction analysis. These interactions may not have occurred.

#### Identification of DDI

An individual case assessment of the 1342 restricted pDDI reports was performed to investigate whether the respective pDDI occurred. In case of a DDI, the causal relationship between the reported DDI and the applied drug pair was assessed by using WHO criteria (The Uppsala Monitoring Centre n.d.). Only reports in which the causal relationship for the drug combination was assessed as “at least possible” were included in the dataset of DDI reports. In that case, “at least possible” means that the causal relationship was either classified as certain, probable/likely, or possible. During the assessment 278 DDI were rated as “at least possible” in 206 reports (hereafter referred to as DDI reports). In 1136 reports the pDDI could not be confirmed (hereafter referred to as ADR reports without DDI).

### Analyses of reports

#### Descriptive analyses

The total dataset of all ADR reports related to psychiatric drugs (*n* = 9276), the pDDI reports (*n* = 1811 reports), the DDI reports (*n* = 206), and the ADR reports without DDI (*n* = 1136) was descriptively analyzed. Thereby, the reports were investigated regarding the demographic parameters and medical histories of the patients, the classification of seriousness, the primary reporting sources, the average number of drugs reported as suspected/interacting or concomitant, the most frequently reported drugs mentioned as suspected/interacting or concomitant and the most common reported ADRs.

The classification of seriousness presents the legal definition (GVP Module VI 2017) and may not correspond to the clinical definition of the severity of an ADR. According to the legal definition, an ADR report is classified as serious, if the ADR was life-threatening, led to hospitalization or prolongation thereof, death, congenital anomalies or permanent disabilities. Notably, more than one seriousness criterion can be reported per ADR report.

The primary reporting source describes the person who created the ADR report (GVP Module VI 2017). One ADR report can be created by more than one reporter (e.g., physician and patient reported independently or together). In the descriptive analysis of the primary reporting source, only the reports created by one reporter are shown.

For the analysis of the most frequently reported ADRs and medical histories of the patients the HLGT level of MedDRA terminology (MedDRA Dictionary n.d.) was used.

#### Output of pDDI according to ABDATA: analysis of pDDI reports

In the 1811 pDDI reports, the average number of pDDI identified by ABDATA was determined (ABDATA Pharma-Daten-Service 2025). Further on, these pDDI were investigated regarding their relevance and plausibility. In ABDATA, the relevance is categorized into severe, moderate, and contraindication and the plausibility into mechanism confirmed, plausible and unknown. Moreover, the three most frequently reported contraindications detected by ADBATA were determined.

#### Output of pDDI according to ABDATA: analysis of restricted pDDI reports

The restricted pDDI reports (*n* = 1342 reports) were grouped by their pDDI according to the output of the ABDATA database (Online Resource 2). Within each pDDI, the most frequently reported potentially interacting drug pairs were evaluated. It should be noted that more than one pDDI could be found in each report. Thus, the number of pDDI may exceed the number of reports. Moreover, the evaluation of pDDI displays the output of the ABDATA interaction analysis. Some of these pDDI summarize more than one symptom or rather describe symptom complexes.

#### Analysis of DDI

During the individual case assessment of the restricted pDDI reports the reported DDI were determined. As some of the pDDI identified by ABDATA were inaccurate summarizing several symptoms, we again grouped the individual DDI identified during the individual case assessment.

### Statistical analyses

Means with standard deviations (SD) and medians with interquartile ranges (IQR) were calculated for patients’ age, the number of drugs and pDDI per ADR report. For all other criteria, frequency distributions were quantified and reported as percentages.

For continuous variables, such as the patient age and the number of drugs reported as suspected/interacting or concomitant, Welch’s two-sample *t*-tests were performed. For categorical variables, chi-square tests or Fisher’s exact tests were used, as appropriate, depending on the sample size.

## Results

### Descriptive analyses of total dataset and pDDI reports

In about 19.5% (*n* = 1811) of all ADR reports related to psychiatric drugs, at least one pDDI between two psychiatric drugs (pDDI reports) could be identified. Regarding the age (51.3 ± 19.0 versus 49.9 ± 18.8, *p*-value 0.003), and sex (females 59.6% versus 61.8%, *p*-value 0.011) of the patients, the distribution was similar in the pDDI reports and the overall dataset of psychiatric drugs (Table 1).

**Table 1:** Descriptive analyses of the overall dataset and the pDDI reports

	Overall dataset (*n* = 9276)^a^	pDDI reports (*n* = 1811)^b^	*p*-value^c^
Demographics of the patients			
Mean Age (± SD)	49.9 (± 18.8)	51.3 (± 19.0)	0.003
Median Age [± IQR]	49.0 [34–63]	50 [36.0–56.0]	
Female (*n*, %)	5733 (61.8%)	1079 (59.6%)	0.011
Male (*n*, %)	3478 (37.5%)	721 (39.8%)	
Unknown (*n*, %)	65 (0.7%)	11 (0.6%)	
Seriousness criteria^d^			
Serious	3650 (39.3%)	1100 (60.7%)	< 0.005
Death	87 (0.9%)	30 (1.7%)	0.009
Life-threatening	267 (2.9%)	100 (5.5%)	< 0.005
Hospitalization	1818 (19.6%)	687 (37.9%)	< 0.005
Disabling	100 (1.1%)	19 (1.0%)	1.000
Number of drugs reported as suspected/interacting or concomitant			
Mean (± SD)	2.8 (± 3.0)	5.3 (± 3.7)	< 0.005
Median [± IQR]	2.0 [1.0–3.0]	4.0 [3.0–7.0]	
The five drugs most frequently reported as suspected/interacting and concomitant (*n*, %)^e^			
1	Venlafaxine (1015, 10.9%)	Quetiapine (418, 23.1%)	-
2	Quetiapine (937, 10.1%)	Mirtazapine (378, 20.9%)	
3	Mirtazapine (769, 8.3%)	Venlafaxine (315, 17.4%)	
4	Risperidone (754, 8.1%)	Risperidone (277, 15.3%)	
5	Levothyroxine (683, 7.4%)	Pantoprazole (250, 14.4%)	
The five ADRs most frequently reported (HLGT level of MedDRA terminology) (*n*, %)^f^			
1	Neurological disorders (1894, 20.4%)	General system disorders (375, 20.7%)	-
2	General system disorders (1859, 20.0%)	Neurological disorders (338, 18.7%)	
3	Therapeutic and non-therapeutic effects (excl toxicity) (1182, 12.7%)	Therapeutic and non-therapeutic effects (excl toxicity) (292, 16.1%)	
4	Gastrointestinal signs and symptoms (1173, 12.6%)	Gastrointestinal signs and symptoms (198, 10.9%)	
5	Movement disorders (incl Parkinsonism) (753, 8.1%)	Movement disorders (incl Parkinsonism) (193, 10.7%)	
The five medical histories most frequently reported (HLGT level of MedDRA terminology) (*n*, %)^f^			
1	Depressed mood disorders and disturbances (1879, 20.3%)	Depressed mood disorders and disturbances (553, 30.5%)	-
2	Schizophrenia and other psychotic disorders (1132, 12.2%)	Schizophrenia and other psychotic disorders (349, 19.3%)	
3	Anxiety disorders and symptoms (1036, 11.2%)	Anxiety disorders and symptoms (224, 12.4%)	
4	Lifestyle issues (1021, 11.0%)	Lifestyle issues (222, 12.3%)	
5	Vascular hypertensive disorders (817, 8.8%)	Vascular hypertensive disorders (218, 12.0%)	
Reporting source^g^			
Physician	2606 (28.1%)	755 (41.7%)	< 0.005
Pharmacist	1222 (13.2%)	249 (13.7%)	0.534
Consumer	4218 (45.5%)	475 (26.2%)	< 0.005
Other HCP	401 (4.3%)	132 (7.3%)	< 0.005

*pDDI* potential drug-drug interaction

^a^The overall dataset contains all ADR reports related to antidepressants, antipsychotics and mood stabilizers (= psychiatric drugs) identified in EudraVigilance

^b^The pDDI reports include all reports with potential DDI identified by ABDATA. In this context, potential does not imply that these DDI actually occurred. Without an individual case assessment, no reliable conclusions can be drawn regarding the occurrence of the pDDI

^c^For continuous variables such as patients age and the number of drugs reported as suspected/interacting or concomitant Welch’s two-sample t-tests were performed. For categorical variables chi-square tests or Fisher’s exact tests were used, as appropriate, depending on the sample size

^d^More than one seriousness criterion can be reported in each ADR report. The evaluation of seriousness follows the legal definition. Thus, an ADR report is classified as serious, if the ADR was life-threatening, led to death, disabilities, congenital anomalies or hospitalization or prolongation thereof

^e^More than one drug can be reported as suspected/interacting or concomitant in an ADR report. The drugs are classified as suspected, interacting or concomitant by the reporter

^f^More than one ADR and medical history can be reported in each ADR report. Note that, MedDRA terminology not only describes ADRs but also conditions, laboratory results, diagnoses and investigations

^g^The primary source qualification describes the person who reported the ADR. More than one primary source qualification can be coded in each ADR report (e.g., physician and consumer reporting about the same ADR). Shown is the number of reports explicitly reported by a physician, pharmacist, consumer or other HCP

Additionally, in both datasets, *depressed mood disorders and disturbances* (30.5% versus 20.3%), *schizophrenia and other psychotic disorders* (19.3% versus 12.2%) and *anxiety disorders and symptoms* (12.4% versus 11.2%) were the most frequently reported medical histories and conditions in the pDDI reports with slightly higher proportions than in the overall dataset.

The pDDI reports were proportionally more often reported by physicians (41.7%, *p*-value < 0.005) and less often by consumers (26.2%, *p*-value < 0.005) compared to the overall dataset (physician 28.2%, consumer 45.5%).

Further on, the pDDI reports were clearly more often designated as serious (60.7% versus 39.3%, *p*-value < 0.005). Likewise, a higher proportion of reports was coded with hospitalization of prolongation thereof (37.9% versus 19.6%, *p*-value < 0.005), life-threatening events (5.5% versus 2.9%, *p*-value < 0.005) and death (1.7% versus 0.9%, *p*-value 0.009).

Considering the ADRs and conditions coded in the pDDI reports, *general system disorders* (375, 20.7%), *neurological disorders* (338, 18.7%), *therapeutic and non-therapeutic effects (excl toxicity)* (292, 16.1%), *gastrointestinal signs and symptoms* (198, 10.9%), and *movement disorders (incl Parkinsonism)* (193, 10.7%) were the most frequently reported.

Table 1 shows the descriptive analysis of the overall data set and the pDDI reports of potentially interacting psychiatric drugs pairs according to ABDATA. The demographical parameters and medical histories of the patients, coding of seriousness criteria, and the drugs most frequently reported as suspected/interacting or concomitant as well as the most common ADRs are shown.

### Output of pDDI according to ABDATA: analysis of pDDI reports

Within the 1811 pDDI reports, 3896 pDDI of 587 different potentially interacting drug pairs were found (Table 2). On average, two pDDI were identified per pDDI report (mean (SD) 2.0 (± 4.1)). About two-thirds of the pDDI were categorized as severe (*n* = 1624, 62.9%). For most of the pDDI the mechanism was either classified as plausible (*n* = 2178, 84.4%) or confirmed (*n* = 214, 8.3%). A total of 9.1% of the pDDI was rated as contraindicated. All three of the most frequently noted contraindications involved clozapine and its potentially increased risk of agranulocytosis and granulocytopenia when combined with lorazepam, amisulpride, or risperidone.

**Table 2:** Output of pDDI according to ABDATA: analysis of pDDI reports

	pDDI reports (*n* = 1811 reports, *n* = 3896 pDDI, *n* = 587 potentially interacting drug pairs)^a^
Number of reported drugs per pDDI report (*n* = 1811)	
Mean (± SD)	5.3 (± 3.7)
Median [± IQR]	4.0 [3.0–7.0]
Number of pDDI per pDDI report (*n* = 1811)	
Mean (± SD)	2.2 (± 4.1)
Median [± IQR]	1.0 [1.0–2.0]
Classification of the pDDI (*n* = 3896) according to ABDATA by their relevance	
Severe	2438 (62.6%)
Moderate severity	1008 (25.9%)
Contraindication	392 (10.1%)
Classification of the pDDI (*n* = 3896) according to ABDATA by their plausibility	
Confirmed mechanism	288 (7.5%)
Plausible mechanism	3342 (85.8%)
Unknown mechanism	266 (6.8%)
The three most frequently identified contraindicated potentially interacting drug pairs (*n* = 392) in the pDDI reports^b^	
1	Clozapine – Lorazepam – increased risk of agranulocytosis and granulocytopenia and unconsciousness, respiratory and cardiac arrest (*n* = 43, 11.0%)
2	Amisulprid – Clozapine – increased risk of agranulocytosis and granulocytopenia (*n* = 42, 10.7%)
3	Clozapine – Risperidone – increased risk of agranulocytosis and granulocytopenia (*n* = 40, 10.2%)

*SD* standard deviation, *IQR* interquartile range, *pDDI* potential drug-drug interaction

^a^The pDDI reports include all reports with potential DDI identified by ABDATA. In this context, potential does not imply that these DDI actually occurred. Without an individual case assessment, no reliable conclusions can be drawn regarding the occurrence of the pDDI

^b^Displayed is the output of the ABDATA database showing the potential drug-drug interactions (pDDI). In this context, potential does not imply that these DDI actually occurred. Additionally, the pDDI can describe one specific symptom or summarize several different symptoms or symptom complexes. Without an individual case assessment, no reliable conclusions can be drawn regarding the occurrence of the pDDI

Table 2 summarizes the output of the pDDI interaction analysis according to ABDATA for the 1811 pDDI reports. Shown is the average number of reported drugs, the average number of pDDI identified by ABDATA and their severity and plausibility, besides the most frequently identified contraindications.

### Output of pDDI according to ABDATA: restricted pDDI reports

**Fig. 2:**
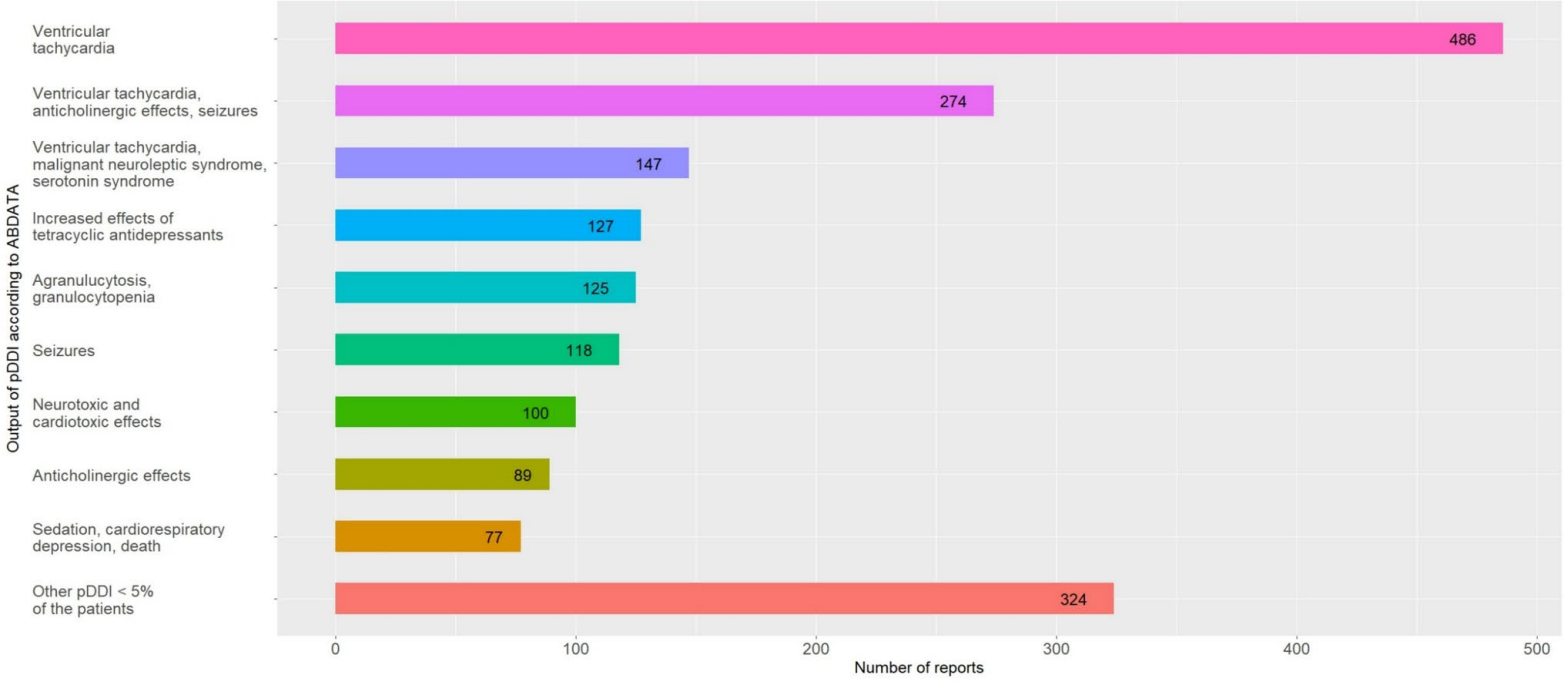
Output of pDDI according to ABDATA: restricted pDDI reports

In the restricted pDDI reports (*n* = 1324), 2343 pDDI were identified. Grouped by pDDI, the pDDI *ventricular tachycardia* (*n* = 486 (36.2%) reports), and the symptom complexes *ventricular tachycardia, anticholinergic effects, seizures* (*n* = 274 (20.4%) reports) and *ventricular tachycardia, extrapyramidal-motoric disorders, malignant neuroleptic syndrome, serotonin syndrome, neuromuscular disorders* (*n* = 147 (11.0%) reports) were the three most frequently identified pDDI according to ABDATA (Fig. 2).

Regarding the pDDI *ventricular tachycardia*, three of the five most frequently reported potentially interacting drug pairs involved quetiapine combined with venlafaxine, sertraline, or escitalopram, and two concerned risperidone combined with venlafaxine or sertraline (Table 3). All five of the most frequently reported potentially interacting drug pairs of the symptom complex *ventricular tachycardia, anticholinergic effects, seizures* comprised mirtazapine, while aripiprazole was entailed in four of the five most commonly reported potentially interacting drug pairs of the symptom complex *ventricular tachycardia, extrapyramidal-motoric disorders, malignant neuroleptic syndrome, serotonin syndrome, neuromuscular disorders*.

**Table 3:** Output of pDDI according to ABDATA: The most frequently detected pDDI and their most frequently reported potentially interacting drugs pairs in the restricted pDDI reports

Rank	pDDI^a^	Potentially interacting drug pairs^a^	Number of reports (% on total number of reports of each potentially interacting drug combination)
1	Ventricular tachycardia	Total pDDI (*n* = 612)	486
		Quetiapine – venlafaxine	85 (17.5%)
		Quetiapine – sertraline	67 (13.8%)
		Quetiapine – escitalopram	45 (9.3%)
		Risperidone – venlafaxine	44 (9.1%)
		Risperidone—sertraline	37 (7.6%)
2	Ventricular tachycardia, anticholinergic effects, seizures	Total pDDI (*n* = 386)	274
		Mirtazapine – quetiapine	78 (28.5%)
		Mirtazapine—venlafaxine	66 (24.1%)
		Mirtazapine – sertraline	50 (18.2%)
		Mirtazapine – risperidone	46 (16.8%)
		Mirtazapine – pipamperone	37 (13.5%)
3	Ventricular tachycardia, extrapyramidal-motoric disorders, malignant neuroleptic syndrome, serotonin syndrome, neuromuscular disorders	Total pDDI (*n* = 170)	147
		Aripiprazole – venlafaxine	33 (22.4%)
		Aripiprazole – sertraline	26 (17.7%)
		Aripiprazole – citalopram	21 (14.3%)
		Amitriptyline – venlafaxine	18 (12.2%)
		Aripiprazole—escitalopram	17 (11.6%)
4	Increased effects of tetracyclic antidepressants	Total pDDI (*n* = 132)	127
		Mirtazapine – citalopram	42 (33.1%)
		Mirtazapine – escitalopram	40 (31.5%)
		Mirtazapine – duloxetine	39 (30.7%)
		Mirtazapine—fluoxetine	11 (8.7%)
5	Agranulocytosis, granulocytopenia	Total pDDI (*n* = 184)	125
		Clozapine – amisulpride	42 (33.6%)
		Clozapine – risperidone	40 (32.0%)
		Clozapine – haloperidol	28 (22.4%)
		Clozapine – valproic acid	24 (19.2%)
		Clozapine—quetiapine	20 (16.0%)
6	Seizures	Total pDDI (*n* = 152)	118
		Bupropion – quetiapine	27 (22.9%)
		Bupropion – venlafaxine	19 (16.1%)
		Bupropion – mirtazapine	16 (13.6%)
		Bupropion – aripiprazole	14 (11.2%)
		Bupropion – escitalopram	14 (11.2%)
7	Neurotoxic and cardiotoxic effects	Total pDDI (*n* = 142)	100
		Lithium – quetiapine	51 (51.0%)
		Lithium – aripiprazole	25 (25.0%)
		Lithium – risperidone	22 (22.0%)
		Lithium – olanzapine	20 (20.0%)
		Lithium – haloperidol	13 (13.0%)
8	Anticholinergic effects	Total pDDI (*n* = 160)	89
		Biperiden – haloperidol	30 (33.7%)
		Biperiden – quetiapine	27 (30.3%)
		Biperiden – risperidone	26 (29.2%)
		Biperiden – paliperidone	19 (21.3%)
		Biperiden – olanzapine	18 (20.2%)
9	Sedation, cardiorespiratory depression, death	Total pDDI (*n* = 80)	77
		Lorazepam – olanzapine	62 (80.5%)
		Diazepam – olanzapine	18 (23.4%)

*pDDI* potential drug-drug interaction

^a^More than pDDI and potentially interacting drug pair may have been identified in one ADR report. Thus, the number of pDDI may exceed the number of reports. The grouping of pDDI follows the output of the ABDATA interaction analysis. Thus, the potential drug-drug interactions (pDDI) are shown. In this context, potential does not imply that these DDI actually occurred. Additionally, the pDDI can describe one specific symptom or summarize several different symptoms or symptom complexes. Without an individual case assessment, no reliable conclusions can be drawn regarding the occurrence of the pDDI

Overall, the five most frequently reported potentially interacting drug pairs were quetiapine–venlafaxine (*n* = 85), mirtazapine–quetiapine (*n* = 78), quetiapine–sertraline (*n* = 67), mirtazapine–venlafaxine (*n* = 66), and lorazepam–olanzapine (*n* = 62) (Online Resource 2).

Figure 2 shows the most frequently identified pDDI according to ABDATA in the restricted pDDI reports. Notably, more than one pDDI could be detected in each ADR report. Thus, one ADR report can be counted for several pDDI but was only counted once per pDDI. Shown are the potential drug-drug interactions (pDDI) of the output of the ABDATA database. In this context, potential does not imply that these DDI actually occurred. Additionally, the pDDI can describe one specific symptom or summarize several different symptoms or symptom complexes. Without an individual case assessment, no reliable conclusions can be drawn regarding the occurrence of the pDDI.

Table 3 presents the nine most frequently identified pDDI according to ABDATA and their five most frequently reported potentially interacting drug pairs in the restricted pDDI reports.

### Identification of DDI

**Fig. 3:**
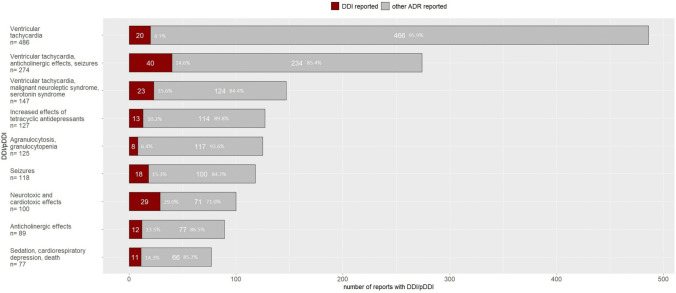
Output of pDDI according to ABDATA: Number of reports with confirmed DDI and other ADRs

We identified 278 DDI (11.9%) in 206 reports (15.4%) in 2343 pDDI in 1342 reports. A DDI was observed in 4.1% (*n* = 20) of the 486 reports of the pDDI *ventricular tachycardia* (Fig. 3). A higher proportion of DDI was found in the reports of the pDDI *ventricular tachycardia, anticholinergic effects, seizures* (14.6%, 40/274 reports), and the reports of the pDDI *increased effects of tetracyclic antidepressants* (15.6%, 23/147). In contrast, the highest proportion of DDI (29.0%) was detected for the pDDI *neurotoxic and cardiotoxic effects* (a more detailed comparative analysis of reports with than without DDI can be found in Online Resource 3). Overall, the five most frequently reported interacting drug pairs were lamotrigine–valproic acid (*n* = 16 reports), mirtazapine–quetiapine (*n* = 14 reports), lithium–quetiapine (*n* = 12 reports), mirtazapine–risperidone (*n* = 11 reports), and lithium–olanzapine (*n* = 10 reports) (Online Resource 4).

Figure 3 shows the number of reports with and without DDI grouped by pDDI according to the ABDATA interaction analysis. Notably, shown is the grouping of the output of the potential drug-drug interactions (pDDI) of the ABDATA database. In this context, potential does not imply that these DDI actually occurred. Additionally, the pDDI can describe one specific symptom or summarize several different symptoms or symptom complexes. Without an individual case assessment, no reliable conclusions can be drawn regarding the occurrence of the pDDI.

### Analysis of DDI reports and ADR reports without DDI

Patients in DDI reports (52.6 (± 18.8)) were only slightly older than patients in ADR reports without DDI (50.4 ± 18.9, *p*-value 0.121) (Table 4). On average more drugs were reported as suspected, interacting or concomitant in the DDI reports (6.0 ± 3.9) compared to those without DDI (5.3 ± 3.7, *p*-value 0.013). Although around two-thirds of ADR reports without DDI (59.0%) were classified as serious, this proportion was even higher among ADR reports with DDI (81.1%, *p*-value < 0.005). A similar pattern was observed for the other seriousness criteria like life-threatening (4.8% (without DDI) versus 10.7% (with DDI), *p*-value 0.001), hospitalization or prolongation thereof (37.5% (without DDI) versus 49.5% (with DDI), *p*-value 0.001), and death (1.1% (without DDI) versus 3.9% (with DDI), *p*-value 0.006).

Less DDI reports were reported by consumers (18.9%) compared to ADR reports without DDI (25.8%, *p*-value 0.044). Patients for whom *schizophrenia and other psychotic disorders* were recorded in the patient’s history were more often contained in the DDI reports (24.8% versus 21.0%). In contrast, patients with a reported history of *depressed mood disorders and disturbances* were more commonly present in ADR reports without DDI (23.8% versus 32.0%).

Table 4 shows the descriptive analysis of the DDI reports and the ADR reports without DDI. The demographical parameters and medical histories of the patients, the coding of seriousness criteria, and the drugs most frequently reported as suspected/interacting of concomitant as well as the most commonly identified interacting drug pairs are shown.

**Table 4:** Descriptive analysis of DDI reports and ADR reports without DDI

	DDI reports (*n* = 206)^a^	ADR reports without DDI (*n* = 1136)^a^	*p*-values^b^
Demographical parameters of the patients			
Mean Age (± SD)	52.6 (± 18.8)	50.4 (± 18.9)	0.121
Median Age [± IQR]	53.0 [40.0–66.0]	49.0 [35.0–63.0]	
Female (*n*, %)	57.8% (*n* = 119)	58.7% (*n* = 667)	0.876
Male (*n*, %)	41.7% (*n* = 86)	40.9% (*n* = 465)	
Unknown (*n*, %)	0.5% (*n* = 1)	0.4% (*n* = 4)	
Seriousness of the ADR reports^c^			
Serious	81.1% (*n* = 167)	59.0% (*n* = 670)	< 0.005
Death	3.9% (*n* = 8)	1.1% (*n* = 12)	0.006
Life-threatening	10.7% (*n* = 22)	4.8% (*n* = 54)	0.001
Hospitalization	49.5% (*n* = 102)	37.5% (*n* = 426)	0.001
Disabling	1.5% (*n* = 3)	0.7% (*n* = 8)	0.231
Primary reporting source^d^			
Physicians	47.1% (*n* = 97)	43.5% (*n* = 494)	0.378
Pharmacists	18.0% (*n* = 37)	12.8% (*n* = 145)	0.058
Other HCP	5.8% (*n* = 12)	7.4% (*n* = 84)	0.511
Non-HCP	18.9% (*n* = 39)	25.8% (*n* = 293)	0.044
Most frequently reported histories of the patients (HLGT level of MedDRA terminology)^e^			
NA	23.3% (*n* = 48)	22.8% (*n* = 259)	-
1	24.8% Schizophrenia and other psychotic disorders (*n* = 51)	32.0% Depressed mood disorders and disturbances (*n* = 363)	
2	23.8% Depressed mood disorders and disturbances (*n* = 49)	21.0% Schizophrenia and other psychotic disorders (*n* = 238)	
3	14.6% Vascular hypertensive disorders (*n* = 30)	13.6% Lifestyle issues (*n* = 155)	
4	13.6% Seizures (incl subtypes) (*n* = 28)	12.6% Anxiety disorders and symptoms (*n* = 143)	
5	10.7% Psychiatric disorders NEC (*n* = 22)	11.8% Vascular hypertensive disorders (*n* = 134)	
Number of drugs reported as suspected, interacting or concomitant			
Mean (± SD)	6.0 (± 3.9)	5.3 (± 3.7)	0.013
Median [± IQR]	5.0 [3.0–8.0]	4.0 [3.0–7.0]	
Most frequently reported drugs (suspected, interacting and concomitant)^e^			
1	29.1% Mirtazapine (*n* = 60)	29.7% Quetiapine (*n* = 337)	-
2	26.2% Valproic acid (*n* = 54)	26.5% Mirtazapine (*n* = 301)	
3	25.7% Quetiapine (*n* = 53)	22.0% Venlafaxine (*n* = 250)	
4	23.3% Risperidone (*n* = 48)	16.8% Risperidone (*n* = 191)	
5	22.3% Lorazepam (*n* = 46)	16.3% Sertraline (*n* = 185)	
Most frequently identified potentially interacting psychiatric drug pairs^e^			
1	7.8% Lamotrigin – valproic acid (*n* = 16)	6.9% Quetiapine – venlafaxine (*n* = 78)	-
2	6.8% Mirtazapine – quetiapine (*n* = 14)	5.1% Mirtazapine – quetiapine (*n* = 58)	
3	5.8% Lithium – quetiapine (*n* = 12)	5.1% Quetiapine – sertraline (*n* = 58)	
4	5.3% Mirtazapine – risperidone (*n* = 11)	4.8% Mirtazapine – venlafaxine (*n* = 55)	
5	4.9% Lithium – olanzapine (*n* = 10)	4.2% Lorazepam – olanzapine (*n* = 48)	

*SD* standard deviation, *IQR* interquartile range, *pDDI* potential drug-drug interaction

^a^The DDI were identified during the individual case assessment of the 1342 restricted pDDI reports

^b^For continuous variables such as patients age and the number of drugs reported as suspected/interacting or concomitant Welch’s two-sample t-tests were performed. For categorical variables, chi-square tests or Fisher’s exact tests were used, as appropriate, depending on the sample size

^c^More than one seriousness criterion can be reported for each ADR report. The evaluation of seriousness follows the legal definition. Thus, an ADR report is classified as serious, if the ADR was life-threatening, led to death, disabilities, congenital anomalies or hospitalization or prolongation thereof

^d^The primary source qualification describes the person who reported the ADR. More than one primary source qualification can be coded in each ADR report (e.g., physician and consumer reporting about the same ADR). Shown is the number of reports containing only one primary source qualification

^e^More than one patient history, more than one drug and more than one potentially interacting drug pair can be reported per ADR report. The displayed drugs most frequently reported as suspected/interacting of concomitant, might not be the drugs that contributed to the interaction

### DDI-stratified analyses

**Fig. 4:**
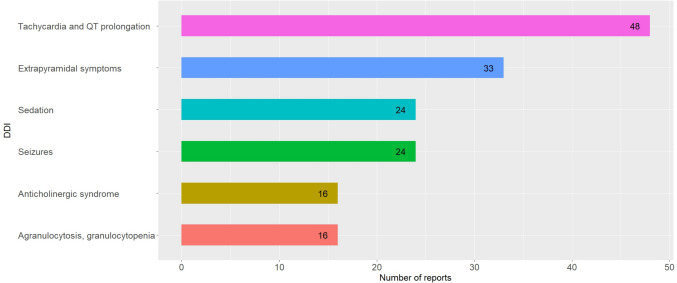
Most frequently identified DDI

Grouped by individual DDI, the most commonly identified DDI was *tachycardia and QT prolongation* (23.3%, 48/206), followed by *extrapyramidal symptoms* (16.0%, 33/206), *sedation* (11.7%, 24/206), and *seizures* (11.7%, 24%206) (Fig. 4, Table 5). Considering interacting drug pairs, the combination mirtazapine–quetiapine (26.3%, 10/48) was most frequently associated with *tachycardia and QT prolongation* (Table 5). Aripiprazole–lithium (18.2%, 6/33) was the interacting drug pair most commonly associated with *extrapyramidal symptoms*, lorazepam–olanzapine (29.2%, 7/24) with *sedation*, and lamotrigine–valproic acid (25.0% 6/24) with *seizures*.

**Table 5:** DDI grouped by individual DDI and their most frequently reported interacting drug pairs

Rank	DDI^a^	Interacting drug pairs^a^	Number of reports (% on total number of reports of each interacting drug combination)
1	Tachycardia and QT prolongation	Total DDI (*n* = 63)	48
		Mirtazapine – quetiapine	10 (26.3%)
		Mirtazapine – venlafaxine	5 (13.2%)
		Aripiprazole – mirtazapine	4 (10.5%)
		Mirtazapine – risperidone	4 (10.5%)
		Citalopram – quetiapine	3 (7.9%)
		Citalopram – risperidone	3 (7.9%)
		Mirtazapine – pipamperone	3 (7.9%)
		Risperidone – venlafaxine	3 (7.9%)
2	Extrapyramidal symptoms	Total DDI (*n* = 48)	33
		Aripiprazole – lithium	6 (18.2%)
		Aripiprazole – sertraline	5 (15.2%)
		Lithium – quetiapine	5 (15.2%)
		Lithium – olanzapine	4 (12.1%)
		Olanzapine – valproic acid	4 (12.1%)
3	Sedation	Total DDI (*n* = 32)	24
		Lorazepam – olanzapine	7 (29.2%)
		Lorazepam – valproic acid	5 (20.8%)
		Diazepam – olanzapine	2 (8.3%)
		Duloxetine – mirtazapine	2 (8.3%)
		Lorazepam – valproic acid	2 (8.3%)
		Mirtazapine – olanzapine	2 (8.3%)
		Mirtazapine – pipamperone	2 (8.3%)
		Mirtazapine – risperidone	2 (8.3%)
4	Seizures	Total DDI (*n* = 30)	24
		Lamotrigine – valproic acid	6 (25.0%)
		Carbamazepine – valproic acid	5 (20.8%)
		Phenobarbital – valproic acid	4 16.7%)
		Carbamazepine – lamotrigine	3 (12.5%)
		Bupropion – escitalopram	2 (8.3%)
		Bupropion – mirtazapine	2 (8.3%)
		Escitalopram – mirtazapine	2 (8.3%)
5	Anticholinergic syndrome	Total DDI (*n* = 21)	16
		Mirtazapine – risperidone	4 (25.0%)
		Biperiden – haloperidol	3 (18.8%)
		Amisulpride – biperiden	2 (12.5%)
		Biperiden – quetiapine	2 (12.5%)
		Biperiden – risperidone	2 (12.5%)
		Melperone – mirtazapine	2 (12.5%)
		Mirtazapine – pipamperone	2 (12.5%)
6	Agranulocytosis, granulocytopenia	Total DDI (*n* = 20)	16
		Clozapine – risperidone	4 (25.0%)
		Clozapine – lorazepam	3 (18.8%)
		Clozapine – quetiapine	3 (18.8%)
		Clozapine – valproic acid	2 (12.5%)
		Quetiapine – valproic acid	2 (12.5%)

^a^More than DDI or interacting drug pair may have been identified in one ADR report. Thus, the number of DDI may exceed the number of reports

Differences in age, sex, comorbidities, and number of drugs used were observed among patients of the six most frequently identified DDI (Table 6). Patients with *tachycardia and QT prolongation* were clearly older (mean 63.0 (± 16.2) years) and took on average 8.0 drugs, while patients with *seizures* (mean 43.5 (± 16.6) years), and *agranulocytosis, granulocytopenia* (mean 42.4 (± 21.1) years) were noticeably younger and took on average 4.6 and 4.2 drugs, respectively. Further on, patients who developed *anticholinergic syndromes were more often* females (75.0%) and patients with *seizures* males (62.5%). All reports of the DDI *agranulocytosis, granulocytopenia* were classified as serious (100.0%). This was also the case for most of the reports describing *tachycardia and QT prolongation* (94.7%), and *seizures* (95.8%), but only for less than two-thirds of the reports describing *extrapyramidal symptoms* (60.6%). Notably, none of the reports of the DDI *agranulocytosis, granulocytopenia* was reported by a consumer. In contrast, this was the case in 30.3% of the reports describing *extrapyramidal symptoms*. Considering the psychiatric disorders reported as a patient’s history, *schizophrenia and other psychotic disorders* were most frequently mentioned in patients who developed *anticholinergic syndromes* (62.5%), *agranulocytosis, granulocytopenia* (50.0%), and *sedation* (33.3%). Compared to that *depressed mood disorders and disturbances* were the patient’s history most evoked for patients who experienced *tachycardia and QT prolongation* (31.6), and *extrapyramidal symptoms* (42.4%), while pre-existing *seizures (incl subtypes)* were reported in more than half of the patients who developed seizures (54.2%). Strikingly, *cardiac arrhythmia* (18.4%) was one of the most recorded patients’ histories in reports describing *tachycardia and QT prolongation*, and *genitourinary tract disorders* (25.0%), and *glaucoma and ocular hypertension* (25.0%) in reports of a*nticholinergic syndromes*.

**Table 6:** DDI-stratified analyses

	Tachycardia and QT prolongation^a^ (*n* = 48 reports, *n* = 63 DDI)	Extrapyramidal symptoms^a^ (*n* = 33 reports, *n* = 48 DDI)	Sedation^a^ (*n* = 24 reports, *n* = 32 DDI)	Seizures^a^ (*n* = 24 reports, *n* = 30 DDI)	Anticholinergic syndrome^a^ (*n* = 16 reports, *n* = 21 DDI)	Agranulocytosis, granulocytopenia^a^ (*n* = 16 reports, *n* = 20 DDI)
Demographical parameters of the patients						
Mean Age (± SD) Median Age [± IQR] Female (*n*, %) Male (*n*, %)	63.0 (± 16.2) 63.5 [51.0–76.8] 63.2% (*n* = 24) 36.8% (*n* = 14)	49.5 (± 15.2) 52.0 [42.0–58.0] 54.5% (*n* = 18) 45.5% (*n* = 15)	57.1 (± 19.5) 57.5 [46.8–68.5] 62.5% (*n* = 15) 33.3% (*n* = 8) 4.2% (*n* = 1)	43.5 (± 16.6) 44.5 [27.8–55.3] 37.5% (*n* = 9) 62.5% (*n* = 15)	51.9 (± 22.0) 44.5 [33.0–76.3] 75.0% (*n* = 12) 25.0% (*n* = 4)	42.4 (± 21.1) 41.5 [22.5–55.0] 56.3% (*n* = 9) 43.8% (*n* = 7)
Seriousness of the ADR reports^b^						
Serious Death Life-threatening Hospitalization Disabling	94.7% (*n* = 36) 5.3% (*n* = 2) 13.2% (*n* = 5) 57.9% (*n* = 22) 2.6% (*n* = 1)	60.6% (*n* = 20) 0.0% (*n* = 0) 0.0% (*n* = 0) 45.5% (*n* = 15) 6.1% (*n* = 2)	83.3% (*n* = 20) 0.0% (*n* = 0) 0.0% (*n* = 0) 54.2% (*n* = 13) 0.0% (*n* = 0)	95.8% (*n* = 23) 0.0% (*n* = 0) 12.5% (*n* = 3) 45.8% (*n* = 11) 0.0% (*n* = 0)	81.3% (*n* = 13) 6.3% (*n* = 1) 12.5% (*n* = 2) 37.5% (*n* = 6) 0.0% (*n* = 0)	100.0% (*n* = 16) 0.0% (*n* = 0) 12.5% (*n* = 2) 68.8% (*n* = 11) 0.0% (*n* = 0)
Primary reporting source^c^						
Physicians Pharmacists Other HCP Non-HCP	44.7% (*n* = 17) 21.1% (*n* = 8) 2.6% (*n* = 1) 18.4% (*n* = 7)	51.5% (*n* = 17) 9.1% (*n* = 3) 3.0% (*n* = 1) 30.3% (*n* = 10)	33.3% (*n* = 8) 29.2% (*n* = 7) 8.3% (*n* = 2) 16.7% (*n* = 4)	33.3% (*n* = 8) 12.5% (*n* = 3) 16.7% (*n* = 4) 25.0% (*n* = 6)	50.0% (*n* = 8) 18.8% (*n* = 3) 12.5% (*n* = 2) 6.3% (*n* = 1)	75.0% (*n* = 12) 18.8% (*n* = 3) 6.3% (*n* = 1) 0.0% (*n* = 0)
Most frequently reported histories of the patients (HLGT level of MedDRA terminology)^d^						
NA 1 2 3 4 5	42.1% (*n* = 16) 31.6% Depressed mood disorders and disturbances (*n* = 12) 18.4% Cardiac arrhythmias (*n* = 7) 18.4% Vascular hypertensive disorders (*n* = 7) 15.8% Psychiatric disorders NEC (*n* = 6) 13.2% Anxiety disorders and symptoms (*n* = 5)	12.1% (*n* = 4) 42.4% Depressed mood disorders and disturbances (*n* = 14) 30.3% Maniac and bipolar mood disorders and disturbances (*n* = 10) 27.3% Schizophrenia and other psychotic disorders (*n* = 9) 21.2% Therapeutic procedures and supportive care (*n* = 7) 15.2% Anxiety disorders and symptoms (*n* = 5) 15.2% Vascular hypertensive disorders (*n* = 5)	29.2% (*n* = 7) 33.3% Schizophrenia and other psychotic disorders (*n* = 8) 16.7% Psychiatric disorders NEC (*n* = 4) 12.5% Bronchial disorders (excl neoplasms) (*n* = 3) 12.5% Lifestyle issues (*n* = 3) 12.5% Mental impairment disorders (*n* = 3) 12.5% Seizures (*n* = 3) 12.5% Vascular hypertensive disorders (*n* = 3)	20.8% (*n* = 5) 54.2% Seizures (incl subtypes) (*n* = 13) 16.7% Therapeutic procedures and supportive care (*n* = 3) 12.5% Nervous system, skull and spine therapeutic procedures (*n* = 3) 12.5% Schizophrenia and other psychotic disorders (*n* = 3) 8.3% Anxiety disorders and symptoms (*n* = 2) 8.3% Glucose metabolism disorders (incl. diabetes mellitus) (*n* = 2) 8.3% Injuries NEC (*n* = 2) 8.3% Mental impairment disorders (*n* = 2) 8.3% Movement disorders (incl Parkinson) (*n* = 2) 8.3% Neurological disorders (*n* = 2) 8.3% Personality disorders and disturbances in behavior (*n* = 2)	12.5% (*n* = 2) 62.5% Schizophrenia and other psychotic disorders (*n* = 10) 31.3% Depressed mood disorders and disturbances (*n* = 5) 25.0% Genitourinary tract disorders (*n* = 4) 25.0% Glaucoma and ocular hypertension (*n* = 4) 25.0% Vascular hypertensive disorders (*n* = 4)	25.0% (*n* = 4) 50.0% Schizophrenia and other psychotic disorders (*n* = 8) 25.0% Psychiatric disorders NEC (*n* = 4) 18.8% Thyroid gland disorders (*n* = 3) 12.5% Movement disorders (incl Parkinson) (*n* = 2) Other (*n* = 1)
Number of drugs reported as suspected, interacting or concomitant						
Mean (± SD) Median [± IQR]	8.0 (± 5.3) 7.0 [4.0–11.0]	4.6 (± 2.0) 4.0 [3.0–6.0]	5.2 (± 2.5) 4.0 [3.8–6.3]	5.5 (± 4.3) 4.0 [3.0–7.0]	6.5 (± 3.1) 5.5 [4.8–8.0]	4.2 (± 2.2) 3.5 [3.0–5.0]
Most frequently reported drugs (suspected, interacting and concomitant)^e^						
1 2 3 4 5	63.2% Mirtazapine (*n* = 24) 44.7% Quetiapine (*n* = 17) 28.9% Pantoprazole (*n* = 11) 26.3% Risperidone (*n* = 10) 26.3% Torasemide (*n* = 10)	45.5% Aripiprazole (*n* = 15) 42.4% Lithium (*n* = 14) 33.3% Quetiapine (*n* = 11) 27.3% Sertraline (*n* = 9) 27.3% Valproic acid (*n* = 9)	54.2% Lorazepam (*n* = 13) 41.7% Olanzapine (*n* = 10) 33.3% Mirtazapine (*n* = 8) 33.3% Valproic acid (*n* = 8) 25.0% Risperidone (*n* = 6)	70.8% Valproic acid (*n* = 17) 50.0% Lamotrigine (*n* = 12) 33.3% Levetiracetam (*n* = 8) 29.2% Carbamazepin (*n* = 7) 20.8% Mirtazapine (*n* = 5)	50.0% Biperiden (*n* = 8) 50.0% Mirtazapine (*n* = 8) 37.5% Ramipril (*n* = 6) 37.5% Risperidone (*n* = 6) 31.3% Bisoprolol (*n* = 5)	75.0% Clozapine (*n* = 12) 37.5% Lorazepam (*n* = 6) 37.5% Quetiapine (*n* = 6) 37.5% Risperidone (*n* = 6) 31.3% Valproic acid (*n* = 5)

^a^The grouping of the DDI follows the individual symptoms reported in the DDI reports. Displayed are the most frequently identified DDI occurring in more than 5% of the reports

^b^More than one seriousness criterion can be reported for each ADR report. The evaluation of seriousness follows the legal definition. Thus, an ADR report is classified as serious, if the ADR was life-threatening, led to death, disabilities, congenital anomalies or hospitalization or prolongation thereof

^c^The primary source qualification describes the person who reported the ADR. More than one primary source qualification can be coded in each ADR report (e.g., physician and consumer reporting about the same ADR). Shown is the number of reports containing only one primary source qualification

^d^More than one medical history can be reported per ADR report. Note that, conditions, laboratory results, diagnoses and investigations can also be coded by using MedDRA terminology

^e^More than one drug or interacting drug pair can be reported per ADR report. The drugs can be classified as suspected, interacting or concomitant by the reporter. The displayed drugs most frequently reported as suspected/interacting of concomitant, might not be the drugs that contributed to the interaction

Figure 4 shows the most frequently identified DDI during the individual case assessment of the 1342 restricted pDDI reports. Notably, more than one DDI could be detected in each ADR report. Thus, one ADR report can be counted for several DDI but was only counted once per DDI. The grouping of DDI follows the individual symptoms reported in the DDI reports. Displayed are the most frequently identified DDI which occurred in more than 5% of the reports.

Table 5 shows the six most frequently identified DDI during the individual case assessment of the 1342 restricted pDDI reports. Notably, more than one DDI could be detected in each ADR report. Thus, one ADR report can be counted for several DDI but was only counted once per DDI. The grouping of the DDI follows the individual symptoms reported in the DDI reports. Displayed are the most frequently identified DDI occurring in more than 5% of the reports. Additionally, only the five most commonly detected interacting drug pairs are shown.

Tab﻿le 6 shows the descriptive analysis of the most frequently identified DDI in the DDI reports grouped by their individual DDI. Notably, more than one DDI could be detected in each ADR report. Thus, one ADR report can be counted for several DDI but was only counted once per DDI. The demographical parameters and medical histories of the patients, the coding of seriousness criteria and the number of drugs reported as suspected/interacting and concomitant as well as the drugs most frequently reported as suspected/interacting of concomitant are shown.

## Discussion

Our analysis showed that in about a fifth of all ADR reports related to psychiatric drugs, a pDDI between two psychiatric drugs could be detected. These pDDI reports were more often designated as serious compared to reports without pDDI and the proportion continued to increase for reports where the DDI was observed. *Tachycardia and QT prolongation* was the DDI most often identified with an even higher proportion of reports classified as serious than for the overall reports on DDI.

### Seriousness and contraindications

As seen in other studies, the number of drugs used seemed to be associated with a higher likelihood of DDI in our study (Wolff et al. 2021). Likewise, more than half of the pDDI were classified as major (life-threatening, intervention required), and about 7.0% as contraindicated in psychiatric patients in India (Sunny et al. 2022). In our study, the proportion of serious reports even rose to 80% in the reports on DDI, however, it should be noted that in reports without DDI, around 60% of the reports were still classified serious.

In our study, clozapine in combination with lorazepam, or risperidone were the most frequently identified contraindicated drug combinations linked to *agranulocytosis and granulocytopenia*. Clozapine-induced agranulocytosis or neutropenia are well-known severe ADRs, estimated to occur in approximately 0.8% of clozapine-treated patients (Mijovic and MacCabe 2020). Nowadays, the prescription of clozapine is restricted to treatment-resistant schizophrenia. Ideally, concomitant therapy with drugs that also have the potential to cause agranulocytosis and granulocytopenia should be avoided. However, in some cases alternatives may not be available or the efficacy of combination therapy might be superior to monotherapy of clozapine (Correll et al. 2009). In most countries blood count monitoring is mandatory for the entire duration of clozapine therapy, enhancing the early detection of agranulocytosis and granulocytopenia thereby reducing fatalities (Mijovic and MacCabe 2020, Referral Leponex and associated names 2002). However, regular monitoring might indirectly also induce more frequent reporting.

### Tachycardia and QT-prolongation

Antipsychotics and antidepressants can prolong ventricular repolarization causing arrhythmias including torsade de point (TdP) and sudden cardiac death (Sala et al. 2005; Zemrak and Kenna 2008). QT interval-prolonging drugs like antipsychotics and combinations of them are frequently used in psychiatry (Hefner et al. 2021). *Tachycardia and QT prolongation* was the most frequently identified DDI in our analysis. Patient-specific risk factors described in literature are female sex, older age, (ischemic) heart diseases and electrolyte disturbances such as hypokalemia and the intake of more than QT interval-prolonging drug (Hefner et al. 2021; Sala et al. 2005; Tisdale et al. 2013). Our study also showed that older age (mean 63.0) and preexisting arrhythmia (18.4%) were among the most frequently reported patient characteristics in patients who developed *tachycardia and QT prolongation*. With respect to older age, this finding may also reflect prescription patterns, as patients treated with more than one QT interval-prolonging drug in a study involving 10 psychiatric hospitals in Germany were older than those receiving one QT interval-prolonging drug (Hefner et al. 2021). These patients were also more often males. As the number of reports describing *tachycardia and QT prolongation* referring to females was higher compared to males in our study, a higher risk for females might be expected. Female sex is a risk factor for TdP according to the Tisdale risk score (Tisdale et al. 2013).

The combinations of mirtazapine with quetiapine, venlafaxine, aripiprazole, or risperidone were the four most frequently identified interacting psychiatric drug pairs within the reports of *tachycardia and QT prolongation* in our study, although all five drugs are classified with a moderate potential of QT prolongation (CredibleMeds). Except for the combination mirtazapine—aripiprazole, these combinations also belonged to the most frequently prescribed QT interval-prolonging drug combinations in the German study mentioned above (Hefner et al. 2021). This could explain their higher number of reports in our study. ECG monitoring is an appropriate tool for monitoring tachycardia and QT prolongation and should be conducted during up-titration and at least yearly afterwards (DGPPN e.V. 2019; Hefner et al. 2021; Sala et al. 2005). Furthermore, TDM, monitoring of electrolyte levels (e.g., hypokalemia), slow up-titration of QT-prolonging drugs, and consideration of patient-specific risk factors may help to reduce the risk of tachycardia and QT prolongation. In clinical practice the website of the Arizona Center for Education and Research on Therapeutics (AZCERT) (CredibelMeds n.d.) or the Tisdale risk score (Md + Calc n.d.; Tisdale et al. 2013) can be used to predict DDI related to QT-prolonging and TdP-triggering drugs taking patient-specific risk factors into account.

### Extrapyramidal symptoms

Lithium combined with antipsychotics is associated with an increased risk of extrapyramidal symptoms as reported in literature (Oberlinghausen 1999). The risk is even higher for first-generation (e.g., haloperidol) than for second-generation antipsychotics like aripiprazole, quetiapine and olanzapine (D’Souza et al. 2025) which were the three drugs most frequently combined with lithium in our study. Second-generation antipsychotics are mainly prescribed today, probably explaining the lower representation of first-generation antipsychotics in our study (Ramin et al. 2025). Although extrapyramidal symptoms are common when two antipsychotics are combined, our analysis primarily identified combinations of antipsychotics with lithium. Extrapyramidal symptoms present with a broad spectrum of symptoms of impaired movement control such as akathisia, muscle stiffness, and Parkinson-like symptoms including tremors (Sanders and Gillig 2012). To reduce the risk of extrapyramidal symptoms, excessive doses of antipsychotics should be avoided, and lithium concentration should be kept in a low-medium range (Oberlinghausen 1999).

### Interaction of lamotrigine and valproic acid

Lamotrigine and valproic acid (VPA), which can both be used as mood stabilizers, were the drug combination most frequently associated with a DDI in our dataset. Both active ingredients are conjugated by uridine diphosphate glucronyltransferase (UGT) (Diaz et al. 2008; English et al. 2012; Sandson et al. 2005). VPA as an inhibitor of UGT 1A4 increases lamotrigine levels resulting in a higher risk of developing confusion, somnolence, and severe skin rashes such as Stevens-Johnson syndrome. On the contrary, lamotrigine mildly induces phase II mechanisms leading to lower concentrations of VPA blood levels potentially causing a loss of therapeutic effect and, thereby, increasing the risk of seizures. Besides UGT, VPA also inhibits CYP2C9 (Diaz et al. 2008). Through both mechanisms, interactions with other target drugs may occur. Carbamazepine as a potent inducer of UGT can affect the concentration of VPA leading to clinically remarkable reductions of concentrations and an increased risk of seizures. This was the second most common combination reported for patients who developed seizures in our study. Consequently, higher doses of VPA might be necessary. Strikingly, slightly more than half of the patients who developed seizures in our study already had a pre-existing history of seizures. We cannot rule out that the respective anti-seizure drugs might have been used for the treatment of seizures or concomitant seizure diseases.

### Translation into clinical practice

Our study highlights the importance of considering DDI when prescribing more than one psychiatric drug, as ADR reports involving DDI were more commonly classified as serious. Especially patient-specific risk factors should be considered in patients treated with QT-prolonging drugs (Hefner et al. 2021). Moreover, DDI can lead to hospitalizations, treatment failures, medical complications, and even increase patient morbidity and mortality (English et al. 2012; Hefner et al. 2021; Sandson et al. 2005) highlighting the importance of obtaining a precise medical history, prescribing appropriate doses, and performing drug-drug interaction checks or TDM (if possible). When adding new drugs to an already existing pharmacotherapy, these should be initiated with a low dose and up-titrated slowly (English et al. 2012). Thereby, drugs with a minimized risk of DDI should be selected. Current lists of potentially interacting drugs, or software systems can be used to avoid inappropriate combinations (English et al. 2012; Kheshti et al. 2016). Additionally, consulting a clinical pharmacist in psychiatric wards may also help to reduce the occurrence of DDI (Hahn et al. 2013, 2018).

For patients, it might be helpful to redeem their drug prescriptions at a single pharmacy, as the pharmacy’s drug interacting monitoring system may help detect pDDI (Sandson et al. 2005).

In the case of new symptoms (e.g., extrapyramidal symptoms), potential DDI should be considered to avoid prescribing cascades. Moreover, special attention should be paid to older patients, patients with liver and kidney diseases, or several comorbidities probably treated with multiple drugs, as these patients carry a higher risk to develop DDI (Hefner et al. 2021; Wolff et al. 2021).

### Strengths and limitations

One of the strengths of analyses performed in spontaneous reporting databases is the inclusion of all types of patients such as patients with severe mental illnesses, or patients taking multiple drugs (Dubrall et al. 2018). The individual case assessment of reports with pDDI evaluating the co-exposure and the causal relationship between the intake of the respective drugs and the occurrence of the ADR/s is another advantage of our analysis.

The unknown amount of underreporting is one of the major limitations of the analyses of spontaneous reports (Hazell and Shakir 2006). It might differ for specific drugs and ADRs (Hasford et al. 2002). Additionally, the number of drug-exposed patients not experiencing an ADR is unknown. As a result, incidences cannot be calculated (Dubrall et al. 2018).

Besides the underreporting, the completeness of documentation may vary between the reports. Especially information regarding concomitant drugs or medical histories of the patients may not be (fully) reported. In the case of concomitant drugs, this would impact our findings, as we analyzed all possible drug-drug combinations per ADR report. Additionally, the dosage of the respective drugs was not considered, which may have already been adjusted to avoid some of the DDI.

Only the ABDATA database was used for the identification of the pDDI. Others observed that the pDDI identified by comparing several databases differed (Hecker et al. 2022; Kontsioti et al. 2022). This was also the case in terms of sensitivity, specificity, and accuracy in another study analyzing eight databases (including ABDATA) commonly used in the German healthcare system (Pauly et al. 2015). Thus, some pDDI may remain undetected.

Our study presents an analysis of already known pDDIs. Unknown or new DDIs were not the subject of this analysis.

## Conclusion

DDI pose a considerable risk to patients treated with several combinations of antidepressant, antipsychotic,s and mood stabilizers. When adding new drugs to an already existing drug therapy regime, drugs with lower potential of DDI should be preferred, and appropriate measures such as TDM, ECG and EEG monitoring, and laboratory tests (e.g., blood counts) should be performed to detect DDI at an early stage. Special attention should be paid to older patients treated with more than one QT interval-prolonging drug.

## Supplementary Information

Below is the link to the electronic supplementary material

ESM 1 (PDF 156 KB)

ESM 2 (PDF 330 KB)

ESM 3 (PDF 267 KB)

ESM 4 (PDF 149 KB)

## Data Availability

The pseudonymised ADR reports from EudraVigilance are not publicly available and cannot be provided upon request due to data protection requirements. In order to fulfill their legal obligations distinct levels of access to EudraVigilance are provided for various stakeholders ([https://www.ema.europa.eu/en/human-regulatory/research-development/pharmacovigilance/eudravigilance/access-eudravigilance-data)). Being one of the competent authorities in Germany, the Federal Institute for Drugs and Medical Devices is granted with the highest level of access covering the individual spontaneous adverse drug reaction (ADR) reports. This access to the individual spontaneous ADR reports from EudraVigilance is not granted to the public and cannot be provided upon request due to data protection requirements. However, a lower level of access is granted to the public thereby enabling researchers to perform the same analysis in EudraVigilance albeit with aggregated data (public access: https://www.adrreports.eu/en/index.html)). For further information regarding the processing of personal data in the context of the operation of EudraVigilance Human we refer to the European Medicines Agency’s Data Protection Notice for EudraVigilance Human.
