# Supplementary material for: Analysis of drug-drug interactions between psychiatric drugs in spontaneous adverse drug reaction reports from EudraVigilance
Source: Naunyn Schmiedebergs Arch Pharmacol. 2026 Jan 22;399(7):9607–26. doi: 10.1007/s00210-025-04956-5 (PMC13152985; doi:10.1007/s00210-025-04956-5)
Supplement: Supplementary file 1 — (PDF 156 KB) [file 210_2025_4956_MOESM1_ESM.pdf]

Title: Analysis of drug-drug interactions between psychiatric drugs in spontaneous adverse drug reaction reports from EudraVigilance

Journal name: Naunyn-Schmiedeberg's Archives of Pharmacology

Authors:

Diana Dubrall[1,2], Patrick Christ[1,2], Miriam Böhme[2], Martina Hahn[3,4,5], Matthias Schmid[1], Catharina Scholl[2]

[1] Institute for Medical Biometry, Informatics and Epidemiology, University Hospital Bonn, Venusberg-Campus 1, 53127 Bonn, Germany

[2] Research Division, Federal Institute for Drugs and Medical Devices (BfArM), Kurt-Georg-Kiesinger-Allee 3, 53175 Bonn, Germany

[3]Department of mental health, varisano hospital Frankfurt Hoechst, Gotenstr. 6-8, 65929 Frankfurt, Germany

[4]Department of psychiatry, psychosomatics and psychotherapy at the university hospital Frankfurt, Heinrich-Hoffmann-Str. 10, 60528 Frankfurt, Germany

[5]Department of pharmacology and clinical pharmacy at the Philipps-University Marburg, Karl-von-Frisch-Strasse 2, 35043 Marburg, Germany

Corresponding author:

Diana Dubrall

Institute for Medical Biometry, Informatics and Epidemiology, University Hospital Bonn, Venusberg-Campus 1, 53127 Bonn, Germany

Federal Institute for Drugs and Medical Devices (BfArM), Bonn, Germany

Kurt-Georg-Kiesinger-Allee 3, 53175 Bonn.

Tel: 0228-99-307-5345

E-mail: Diana.Dubrall@bfarm.de

Online resource 1) List of drugs for identification of spontaneous ADR reports related to antidepressants, antipsychotics and mood stabilizers.

**Drugs**	**ATC code**
Levomepromazine	N05AA02
Fluphenazine	N05AB02
Perphenazine	N05AB03
Perazine	N05AB10
Thioridazine	N05AC02
Haloperidol	N05AD01
Melperone	N05AD03
Pipamperone	N05AD05
Bromperidol	N05AD06
Benperidol	N05AD07
Sterindole	N05AE03
Ziprasidone	N05AE04
Lurasidone	N05AE05
Flupentixol	N05AF01
Chlorprothixene	N05AF03
Zuclopenthixol	N05AF05
Fluspirilene	N05AG01
Pimozide	N05AG02
Loxapine	N05AH01
Clozapine	N05AH02

Olanzapine	N05AH03
Quetiapine	N05AH04
Asenapine	N05AH05
Sulpiride	N05AL01
Tiapride	N05AL03
Amisulpride	N05AL05
Lithium	N05AN01
Prothipendyl	N05AX07
Risperidone	N05AX08
Aripiprazole	N05AX12
Dehydroaripiprazole	N05AX12
Paliperidone	N05AX13
Cariprazine	N05AX15
Brexpiprazole	N05AX16
Imipramine	N06AA02
Trimipramine	N06AA06
Imipraminoxide	N06AA03
Clomipramine	N06AA04
Desmethylclomipramine	N06AA04
Opipramol	N06AA05
Amitriptyline	N06AA09
Nortriptyline	N06AA10
Desmethyldoxepin	N06AA12
Doxepin	N06AA12
Dosulepin	N06AA16
Maprotiline	N06AA21

Fluoxetine	N06AB03
Norfluoxetine	N06AB03
Citalopram	N06AB04
Desmethylcitalopram	N06AB04
Escitalopram	N06AB10
Paroxetine	N06AB05
Desmethylsertraline	N06AB06
Sertraline	N06AB06
Fluvoxamine	N06AB08
Tranylcypromine	N06AF04
Moclobemide	N06AG02
Mianserin	N06AX03
Trazodone	N06AX05
Mirtazapine	N06AX11
Tianeptine	N06AX14
Venlafaxine	N06AX16
Milnacipran	N06AX07
Duloxetine	N06AX21
Bupropion	N06AX12
Agomelatine	N06AX22
Carbamazepine	N03AF01*
Oxcarbamazepine	N03AF02*
Valproinic acid	N03AG01*
Lamotrigine	N03AX09*

* active substances used as mood stabilizers in clinical practice but not assigned to the ATC classes of antidepressants and antipsychotics.

Online resource 1 shows the drugs and their ATC-code used for the identification of the spontaneous ADR reports in EudraVigilance. These drugs had to be reported as suspected/interacting.
