# Supplementary material for: Analysis of drug-drug interactions between psychiatric drugs in spontaneous adverse drug reaction reports from EudraVigilance
Source: Naunyn Schmiedebergs Arch Pharmacol. 2026 Jan 22;399(7):9607–26. doi: 10.1007/s00210-025-04956-5 (PMC13152985; doi:10.1007/s00210-025-04956-5)
Supplement: Supplementary file 2 — (PDF 330 KB) [file 210_2025_4956_MOESM2_ESM.pdf]

Title: Analysis of drug-drug interactions between psychiatric drugs in spontaneous adverse drug reaction reports from EudraVigilance

Journal name: Naunyn-Schmiedeberg's Archives of Pharmacology

Authors:

Diana Dubrall[1,2], Patrick Christ[1,2], Miriam Böhme[2], Martina Hahn[3,4,5], Matthias Schmid[1], Catharina Scholl[2]

[1] Institute for Medical Biometry, Informatics and Epidemiology, University Hospital Bonn, Venusberg-Campus 1, 53127 Bonn, Germany

[2] Research Division, Federal Institute for Drugs and Medical Devices (BfArM), Kurt-Georg-Kiesinger-Allee 3, 53175 Bonn, Germany

[3]Department of mental health, varisano hospital Frankfurt Hoechst, Gotenstr. 6-8, 65929 Frankfurt, Germany

[4]Department of psychiatry, psychosomatics and psychotherapy at the university hospital Frankfurt, Heinrich-Hoffmann-Str. 10, 60528 Frankfurt, Germany

[5]Department of pharmacology and clinical pharmacy at the Philipps-University Marburg, Karl-von-Frisch-Strasse 2, 35043 Marburg, Germany

Corresponding author:

Diana Dubrall

Institute for Medical Biometry, Informatics and Epidemiology, University Hospital Bonn, Venusberg-Campus 1, 53127 Bonn, Germany

Federal Institute for Drugs and Medical Devices (BfArM), Bonn, Germany

Kurt-Georg-Kiesinger-Allee 3, 53175 Bonn.

Tel: 0228-99-307-5345

E-mail: Diana.Dubrall@bfarm.de

Online Resource 2) List of potentially interacting drug combinations identified by ABDATA.

Potentially interacting drug combinations	Number of reports
Quetiapine - Venlafaxine	85
Mirtazapine - Quetiapine	78
Quetiapine - Sertraline	67
Mirtazapine - Venlafaxine	66
Lorazepam - Olanzapine	62
Lamotrigine - Valproic acid	54
Lithium - Quetiapine	51
Mirtazapine - Sertraline	50
Mirtazapine - Risperidone	46
Escitalopram - Quetiapine	45
Risperidone - Venlafaxine	44
Clozapine - Lorazepam	43
Quetiapine - Valproic acid	43
Amisulpride - Clozapine	42
Citalopram - Mirtazapine	42
Clozapine - Risperidone	40
Escitalopram - Mirtazapine	40
Duloxetine - Mirtazapine	39
Olanzapine - Valproic acid	38
Mirtazapine - Pipamperone	37

Risperidone - Sertraline	37
Lithium - Venlafaxine	36
Mirtazapine - Olanzapine	35
Aripiprazole - Venlafaxine	33
Lorazepam - Valproic acid	33
Biperiden - Haloperidol	30
Citalopram - Quetiapine	30
Citalopram - Risperidone	28
Clozapine - Haloperidol	28
Aripiprazole - Mirtazapine	27
Biperiden - Quetiapine	27
Bupropion - Quetiapine	27
Aripiprazole - Sertraline	26
Biperiden - Risperidone	26
Aripiprazole - Lithium	25
Clozapine - Valproic acid	24
Melperone - Mirtazapine	24
Lithium - Mirtazapine	22
Lithium - Risperidone	22
Olanzapine - Venlafaxine	22
Aripiprazole - Citalopram	21
Lithium - Sertraline	21
Carbamazepine - Valproic acid	20
Clozapine - Quetiapine	20
Lithium - Olanzapine	20
Olanzapine - Sertraline	20

Pipamperone - Venlafaxine	20
Biperiden - Paliperidone	19
Bupropion - Venlafaxine	19
Escitalopram - Risperidone	19
Pipamperone - Sertraline	19
Promethazine - Venlafaxine	19
Amitriptyline - Venlafaxine	18
Biperiden - Olanzapine	18
Carbamazepine - Lamotrigine	18
Diazepam - Olanzapine	18
Aripiprazole - Escitalopram	17
Amitriptyline - Duloxetine	16
Aripiprazole - Fluoxetine	16
Bupropion - Mirtazapine	16
Clozapine - Paliperidone	16
Duloxetine - Risperidone	16
Levetiracetam - Quetiapine	16
Amitriptyline - Sertraline	15
Citalopram - Olanzapine	15
Levetiracetam - Mirtazapine	15
Promethazine - Sertraline	15
Aripiprazole - Bupropion	14
Biperiden - Clozapine	14
Biperiden - Flupentixol	14
Bupropion - Escitalopram	14
Clozapine - Escitalopram	14

Clozapine - Flupentixol	14
Clozapine - Venlafaxine	14
Haloperidol - Lithium	13
Haloperidol - Venlafaxine	13
Levetiracetam - Risperidone	13
Opipramol - Sertraline	13
Amisulpride - Biperiden	12
Escitalopram - Olanzapine	12
Mirtazapine - Promethazine	12
Quetiapine - Trazodone	12
TrImipramine - Venlafaxine	12
Amitriptyline - Quetiapine	11
Clozapine - Lithium	11
Clozapine - Sertraline	11
Escitalopram - TrImipramine	11
Fluoxetine - Mirtazapine	11
Melperone - Sertraline	11
Phenobarbital - Valproic acid	11
Amisulpride - Venlafaxine	10
Aripiprazole - Carbamazepine	10
Bupropion - Sertraline	10
Clobazam - Valproic acid	10
Clozapine - Lamotrigine	10
Clozapine - Melperone	10
Escitalopram - Promethazine	10
Fluoxetine - Quetiapine	10

Lamotrigine - Oxcarbazepine	10
Levetiracetam - Pipamperone	10
Levetiracetam - Venlafaxine	10
Mirtazapine - Prothipendyl	10
Amisulpride - Mirtazapine	9
Amitriptyline - Escitalopram	9
Bupropion - Citalopram	9
Carbamazepine - Quetiapine	9
Carbamazepine - Risperidone	9
Citalopram - Clozapine	9
Clozapine - Paroxetine	9
Donepezil - Risperidone	9
Doxepine - Sertraline	9
Levetiracetam - Melperone	9
Melperone - Venlafaxine	9
Phenytoin - Valproic acid	9
Quetiapine - TrImipramine	9
Amitriptyline - Carbamazepine	8
Amitriptyline - Lithium	8
Bupropion - Pipamperone	8
Carbamazepine - Olanzapine	8
Citalopram - Doxepine	8
Citalopram - Pipamperone	8
Clozapine - Mirtazapine	8
Donepezil - Mirtazapine	8
Doxepine - Risperidone	8

Escitalopram - Levetiracetam	8
Haloperidol - Mirtazapine	8
Lithium - Pipamperone	8
Mirtazapine - Paroxetine	8
Opipramol - Quetiapine	8
Pipamperone - Trazodone	8
Pipamperone - TrImipramine	8
Amisulpride - Citalopram	7
Amisulpride - Escitalopram	7
Amitriptyline - Aripiprazole	7
Aripiprazole - Trazodone	7
Biperiden - Zuclopenthixol	7
Bupropion - Duloxetine	7
Bupropion - Fluoxetine	7
Bupropion - Olanzapine	7
Carbamazepine - Haloperidol	7
Carbamazepine - Mirtazapine	7
Citalopram - Melperone	7
Citalopram - Opipramol	7
Citalopram - Paliperidone	7
Citalopram - TrImipramine	7
Clozapine - Diazepam	7
Clozapine - Zuclopenthixol	7
Duloxetine - Trazodone	7
Flupentixol - Lithium	7
Olanzapine - Trazodone	7

Olanzapine - TrImipramine	7
Opipramol - Venlafaxine	7
Primidone - Valproic acid	7
Prothipendyl - Sertraline	7
Prothipendyl - Venlafaxine	7
Amitriptyline - Prothipendyl	6
Amitriptyline - Risperidone	6
Aripiprazole - Paroxetine	6
Biperiden - Melperone	6
Biperiden - Pipamperone	6
Bupropion - Doxepine	6
Bupropion - Promethazine	6
Bupropion - Risperidone	6
Chlorprothixen - Sertraline	6
Citalopram - Duloxetine	6
Citalopram - Haloperidol	6
Citalopram - Levetiracetam	6
Citalopram - Lithium	6
Citalopram - Promethazine	6
Clomipramine - Quetiapine	6
Clozapine - Fluvoxamine	6
Clozapine - Promethazine	6
Donepezil - Quetiapine	6
Doxepine - Duloxetine	6
Doxepine - Promethazine	6
Doxepine - Venlafaxine	6

Duloxetine - Opipramol	6
Duloxetine - Venlafaxine	6
Escitalopram - Lithium	6
Escitalopram - Opipramol	6
Fluoxetine - Risperidone	6
Haloperidol - Sertraline	6
Lamotrigine - Phenytoin	6
Lithium - Paliperidone	6
Melperone - TrImipramine	6
Mirtazapine - Tranylcypromine	6
Paliperidone - TrImipramine	6
Paliperidone - Venlafaxine	6
Amisulpride - Lithium	5
Amisulpride - Sertraline	5
Amitriptyline - Citalopram	5
Amitriptyline - Haloperidol	5
Amitriptyline - Melperone	5
Aripiprazole - Levetiracetam	5
Aripiprazole - TrImipramine	5
Bupropion - Clozapine	5
Bupropion - Methylphenidate	5
Bupropion - Prothipendyl	5
Carbamazepine - Clobazam	5
Carbamazepine - Diazepam	5
Chlorprothixen - Mirtazapine	5
Citalopram - Prothipendyl	5

Doxepine - Pipamperone	5
Duloxetine - Milnacipran	5
Escitalopram - Haloperidol	5
Escitalopram - Pipamperone	5
Escitalopram - Prothipendyl	5
Fluoxetine - Promethazine	5
Haloperidol - Levetiracetam	5
Lamotrigine - Primidone	5
Levetiracetam - Olanzapine	5
Lithium - Milnacipran	5
Lithium - Promethazine	5
Lithium - Trazodone	5
Mirtazapine - Perazine	5
Olanzapine - Paroxetine	5
Opipramol - Risperidone	5
Risperidone - Trazodone	5
Risperidone - TrImipramine	5
Amisulpride - Bupropion	4
Amisulpride - Fluoxetine	4
Amitriptyline - Fluoxetine	4
Amitriptyline - Olanzapine	4
Amitriptyline - Pipamperone	4
Aripiprazole - Donepezil	4
Biperiden - Perazine	4
Bupropion - Melperone	4
Bupropion - Opipramol	4

Bupropion - TrImipramine	4
Bupropion - Valproic acid	4
Carbamazepine - Fluoxetine	4
Carbamazepine - Phenytoin	4
Carbamazepine - Zonisamide	4
Citalopram - Flupentixol	4
Clozapine - Levetiracetam	4
Doxepine - Melperone	4
Doxepine - Olanzapine	4
Duloxetine - Escitalopram	4
Duloxetine - Fluoxetine	4
Escitalopram - Flupentixol	4
Escitalopram - Melperone	4
Eslicarbazepine - Lamotrigine	4
Fluoxetine - Olanzapine	4
Imipramine - Olanzapine	4
Lamotrigine - Phenobarbital	4
Levomepromazine - Sertraline	4
Lisdexamfetamine - Venlafaxine	4
Lithium - Melperone	4
Lithium - Zuclopenthixol	4
Methylphenidate - Venlafaxine	4
Opipramol - Promethazine	4
Paliperidone - Sertraline	4
Pramipexol - Quetiapine	4
Promethazine - Trazodone	4

Sertraline - TrImipramine	4
Amitriptyline - Bupropion	3
Amitriptyline - Clozapine	3
Amitriptyline - Paroxetine	3
Amitriptyline - Promethazine	3
Amitriptyline - Tranylcypromine	3
Amitriptyline - Valproic acid	3
Asenapin - Escitalopram	3
Bupropion - Carbamazepine	3
Bupropion - Clomipramine	3
Bupropion - Milnacipran	3
Bupropion - Tranylcypromine	3
Cannabidiol - Valproic acid	3
Carbamazepine - Clonazepam	3
Carbamazepine - Clozapine	3
Chlorprothixen - Citalopram	3
Chlorprothixen - Escitalopram	3
Chlorprothixen - Venlafaxine	3
Citalopram - Fampridine	3
Citalopram - Perazine	3
Citalopram - Tranylcypromine	3
Citalopram - Ziprasidone	3
Clobazam - Paroxetine	3
Clomipramine - Venlafaxine	3
Clonazepam - Olanzapine	3
Clozapine - Fluoxetine	3

Donepezil - Melperone	3
Doxepine - Haloperidol	3
Duloxetine - Lisdexamfetamine	3
Escitalopram - Fluvoxamine	3
Escitalopram - Paliperidone	3
Escitalopram - Perazine	3
Ethosuximid - Valproic acid	3
Fampridine - Venlafaxine	3
Fluoxetine - Perazine	3
Fluoxetine - TrImipramine	3
Flupentixol - Sertraline	3
Fluvoxamine - Haloperidol	3
Levomepromazine - Lithium	3
Levomepromazine - Venlafaxine	3
Lithium - Nortriptyline	3
Lithium - Paroxetine	3
Melperone - Trazodone	3
Methadone - Pipamperone	3
Methylphenidate - Risperidone	3
Mirtazapine - Sulpiride	3
Mirtazapine - Zuclopenthixol	3
Moclobemide - Rasagiline	3
Nortriptyline - Quetiapine	3
Oxcarbazepine - Perampanel	3
Paroxetine - Risperidone	3
Perazine - Venlafaxine	3

Prothipendyl - Trazodone	3
Sertraline - Ziprasidone	3
Amisulpride - Amitriptyline	2
Amisulpride - Doxepine	2
Amisulpride - Trazodone	2
Amitriptyline - Biperiden	2
Amitriptyline - Chlorprothixen	2
Amitriptyline - Flupentixol	2
Amitriptyline - Perazine	2
Aripiprazole - Doxepine	2
Aripiprazole - Methadone	2
Aripiprazole - Opipramol	2
Aripiprazole - Tetrabenazine	2
Atomoxetine - Citalopram	2
Atomoxetine - Levomepromazine	2
Atomoxetine - Quetiapine	2
Benperidol - Biperiden	2
Biperiden - Levomepromazine	2
Biperiden - Ziprasidone	2
Bupropion - Donepezil	2
Bupropion - Haloperidol	2
Bupropion - Nortriptyline	2
Bupropion - Paroxetine	2
Bupropion - Trazodone	2
Carbamazepine - Doxepine	2
Carbamazepine - Perampanel	2

Chlorprothixen - Clozapine	2
Chlorprothixen - Doxepine	2
Chlorprothixen - Lithium	2
Clobazam - Fluoxetine	2
Clomipramine - Lithium	2
Clomipramine - Sertraline	2
Clonazepam - Clozapine	2
Clozapine - Oxazepam	2
Clozapine - Oxcarbazepine	2
Clozapine - Perazine	2
Clozapine - Perphenazine	2
Clozapine - Trazodone	2
Clozapine - TrImipramine	2
Clozapine - Zonisamide	2
Donepezil - Haloperidol	2
Donepezil - Levomepromazine	2
Donepezil - Pipamperone	2
Donepezil - Venlafaxine	2
Doxepine - Fluoxetine	2
Doxepine - Flupentixol	2
Doxepine - Methadone	2
Doxepine - Paliperidone	2
Duloxetine - Moclobemide	2
Duloxetine - Paroxetine	2
Duloxetine - Sertraline	2
Duloxetine - Tranylcypromine	2

Escitalopram - Levomepromazine	2
Escitalopram - Zuclopenthixol	2
Fampridine - Sertraline	2
Fluoxetine - Lithium	2
Fluoxetine - Melperone	2
Fluoxetine - Valproic acid	2
Fluvoxamine - Mirtazapine	2
Fluvoxamine - Olanzapine	2
Fluvoxamine - Quetiapine	2
Haloperidol - Paroxetine	2
Haloperidol - Trazodone	2
Imipramine - Quetiapine	2
Imipramine - Risperidone	2
Levetiracetam - Trazodone	2
Lithium - Perazine	2
Lithium - TrImipramine	2
Methadone - Olanzapine	2
Methylphenidate - Nortriptyline	2
Mianserine - Venlafaxine	2
Milnacipran - Tranylcypromine	2
Mirtazapine - Moclobemide	2
Mirtazapine - Paliperidone	2
Nortriptyline - Venlafaxine	2
Opipramol - Pipamperone	2
Opipramol - Prothipendyl	2
Opipramol - Sulpiride	2

Oxcarbazepine - Phenytoin	2
Paroxetine - TrImipramine	2
Perazine - Sertraline	2
Phenytoin - Risperidone	2
Quetiapine - Tetrabenazine	2
Sulpiride - TrImipramine	2
Tiapride - Venlafaxine	2
Tranylcypromine - Trazodone	2
TrImipramine - Valproic acid	2
Alprazolam - Clozapine	1
Amfetamin - Sertraline	1
Amisulpride - Clomipramine	1
Amisulpride - Imipramine	1
Amisulpride - Levetiracetam	1
Amisulpride - Opipramol	1
Amisulpride - Trihexyphenidyl	1
Amisulpride - TrImipramine	1
Amitriptyline - Bornaprine	1
Amitriptyline - Fampridine	1
Amitriptyline - Levetiracetam	1
Amitriptyline - Levomepromazine	1
Amitriptyline - Methylphenidate	1
Amitriptyline - Paliperidone	1
Amitriptyline - Perphenazine	1
Amitriptyline - Sulpiride	1
Amitriptyline - Thioridazine	1

Amitriptylineoxide - Lithium	1
Amitriptylineoxide - Tranylcypromine	1
Amitriptylineoxide - Venlafaxine	1
Aripiprazole - Clomipramine	1
Aripiprazole - Imipramine	1
Aripiprazole - Nortriptyline	1
Aripiprazole - Tiapride	1
Asenapin - Biperiden	1
Atomoxetine - Bupropion	1
Atomoxetine - Sertraline	1
Atomoxetine - Venlafaxine	1
Benperidol - Sertraline	1
Biperiden - Chlorprothixen	1
Biperiden - Doxepine	1
Biperiden - Fluphenazine	1
Biperiden - Imipramine	1
Biperiden - Perphenazine	1
Bromazepam - Carbamazepine	1
Bromperidol - Venlafaxine	1
Bupropion - Flupentixol	1
Bupropion - Lisdexamfetamine	1
Bupropion - Perazine	1
Bupropion - Pitolisant	1
Bupropion - Sulpiride	1
Bupropion - Vortioxetine	1
Carbamazepine - Clomipramine	1

Carbamazepine - Flunitrazepam	1
Carbamazepine - Imipramine	1
Carbamazepine - Lithium	1
Carbamazepine - Midazolam	1
Carbamazepine - Moclobemide	1
Carbamazepine - Nortriptyline	1
Carbamazepine - Sertraline	1
Carbamazepine - TrImipramine	1
Carbamazepine - Vortioxetine	1
Carbamazepine - Zopiclone	1
Chlorprothixen - Clomipramine	1
Chlorprothixen - Fampridine	1
Chlorprothixen - Methadone	1
Chlorprothixen - Opipramol	1
Chlorprothixen - Trazodone	1
Citalopram - Clomipramine	1
Citalopram - Donepezil	1
Citalopram - Levomepromazine	1
Citalopram - Moclobemide	1
Citalopram - Sertindol	1
Citalopram - Zuclopenthixol	1
Clobazam - Clozapine	1
Clomipramine - Clozapine	1
Clomipramine - Escitalopram	1
Clomipramine - Fluoxetine	1
Clomipramine - Flupentixol	1

Clomipramine - Haloperidol	1
Clomipramine - Levetiracetam	1
Clomipramine - Levomepromazine	1
Clomipramine - Methylphenidate	1
Clomipramine - Paroxetine	1
Clomipramine - Perazine	1
Clomipramine - Promethazine	1
Clomipramine - Prothipendyl	1
Clomipramine - Risperidone	1
Clomipramine - Valproic acid	1
Clozapine - Doxepine	1
Clozapine - Imipramine	1
Clozapine - Pramipexol	1
Clozapine - Primidone	1
Clozapine - Ropinirol	1
Clozapine - Sertindol	1
Donepezil - Escitalopram	1
Donepezil - Flupentixol	1
Donepezil - Sulpiride	1
Doxepine - Escitalopram	1
Doxepine - Levomepromazine	1
Doxepine - Methylphenidate	1
Doxepine - Paroxetine	1
Doxepine - Phenytoin	1
Doxepine - Prothipendyl	1
Doxepine - Sulpiride	1

Duloxetine - Fluvoxamine	1
Duloxetine - Rasagiline	1
Duloxetine - Safinamide	1
Escitalopram - Fampridine	1
Escitalopram - Imipramine	1
Escitalopram - St. John’s wort	1
Escitalopram - Nortriptyline	1
Fampridine - Fluoxetine	1
Fampridine - Olanzapine	1
Fampridine - Zuclopenthixol	1
Fluoxetine - Haloperidol	1
Fluoxetine - Levomepromazine	1
Fluoxetine - Moclobemide	1
Fluoxetine - Nortriptyline	1
Fluoxetine - Opipramol	1
Fluoxetine - Paliperidone	1
Fluoxetine - Prothipendyl	1
Fluoxetine - Tryptophane	1
Fluoxetine - Vortioxetine	1
Flupentixol - Mirtazapine	1
Flupentixol - Paroxetine	1
Flupentixol - Trazodone	1
Flupentixol - Venlafaxine	1
Fluphenazine - Imipramine	1
Fluvoxamine - Risperidone	1
Haloperidol - Imipramine	1

Haloperidol - Methadone	1
Haloperidol - Nortriptyline	1
Haloperidol - Tetrabenazine	1
Imipramine - Moclobemide	1
Imipramine - Pipamperone	1
Imipramine - Promethazine	1
Imipramine - Valproic acid	1
Imipramine - Venlafaxine	1
St. John’s wort - Milnacipran	1
Levetiracetam - Lithium	1
Levetiracetam - Nortriptyline	1
Levetiracetam - Paliperidone	1
Levetiracetam - Sulpiride	1
Levetiracetam - Zuclopenthixol	1
Levomepromazine - Mianserine	1
Levomepromazine - Mirtazapine	1
Levomepromazine - Opipramol	1
Levomepromazine - Rotigotine	1
Levomepromazine - Trazodone	1
Lisdexamfetamine - Trazodone	1
Lithium - Oxcarbazepine	1
Lithium - Thioridazine	1
Lithium - Vortioxetine	1
Lithium - Ziprasidone	1
Melperone - Methadone	1
Melperone - Opipramol	1

Methadone - Milnacipran	1
Methadone - Mirtazapine	1
Methadone - Quetiapine	1
Methylphenidate - Milnacipran	1
Methylphenidate - Mirtazapine	1
Mianserine - Prothipendyl	1
Mianserine - Quetiapine	1
Mianserine - Sertraline	1
Mianserine - Ziprasidone	1
Mirtazapine - Primidone	1
Mirtazapine - Rasagiline	1
Mirtazapine - Ziprasidone	1
Moclobemide - Trazodone	1
Nortriptyline - Olanzapine	1
Nortriptyline - Paroxetine	1
Nortriptyline - Perazine	1
Nortriptyline - Promethazine	1
Nortriptyline - Sertraline	1
Nortriptyline - Thioridazine	1
Nortriptyline - Valproic acid	1
Olanzapine - Opipramol	1
Olanzapine - Sertindol	1
Olanzapine - Tetrabenazine	1
Opipramol - Paroxetine	1
Opipramol - Primidone	1
Paliperidone - Trihexyphenidyl	1

Paroxetine - Perphenazine	1
Paroxetine - Promethazine	1
Paroxetine - Tryptophane	1
Paroxetine - Vortioxetine	1
Paroxetine - Zuclopenthixol	1
Perazine - Tiapride	1
Perphenazine - Venlafaxine	1
Phenobarbital - Risperidone	1
Primidone - Risperidone	1
Quetiapine - Ropinirol	1
Quetiapine - Rotigotine	1
Quetiapine - Tiapride	1
Rasagiline - Trazodone	1
Risperidone - Rotigotine	1
Rufinamide - Valproic acid	1
Sertraline - Tryptophane	1
Sertraline - Zuclopenthixol	1
Tetrabenazine - Tiapride	1
Tianeptine - Valproic acid	1
Tranylcypromine - TrImipramine	1
Venlafaxine - Ziprasidone	1

Online Resource 2 lists all potentially interacting drug pairs identified by the ABDATA interaction analysis.
