# Supplementary material for: Analysis of drug-drug interactions between psychiatric drugs in spontaneous adverse drug reaction reports from EudraVigilance
Source: Naunyn Schmiedebergs Arch Pharmacol. 2026 Jan 22;399(7):9607–26. doi: 10.1007/s00210-025-04956-5 (PMC13152985; doi:10.1007/s00210-025-04956-5)
Supplement: Supplementary file 3 — (PDF 267 KB) [file 210_2025_4956_MOESM3_ESM.pdf]

Title: Analysis of drug-drug interactions between psychiatric drugs in spontaneous adverse drug reaction reports from EudraVigilance

Journal name: Naunyn-Schmiedeberg's Archives of Pharmacology

Authors:

Diana Dubrall[1,2], Patrick Christ[1,2], Miriam Böhme[2], Martina Hahn[3,4,5], Matthias Schmid[1], Catharina Scholl[2]

[1] Institute for Medical Biometry, Informatics and Epidemiology, University Hospital Bonn, Venusberg-Campus 1, 53127 Bonn, Germany

[2] Research Division, Federal Institute for Drugs and Medical Devices (BfArM), Kurt-Georg-Kiesinger-Allee 3, 53175 Bonn, Germany

[3]Department of mental health, varisano hospital Frankfurt Hoechst, Gotenstr. 6-8, 65929 Frankfurt, Germany

[4]Department of psychiatry, psychosomatics and psychotherapy at the university hospital Frankfurt, Heinrich-Hoffmann-Str. 10, 60528 Frankfurt, Germany

[5]Department of pharmacology and clinical pharmacy at the Philipps-University Marburg, Karl-von-Frisch-Strasse 2, 35043 Marburg, Germany

Corresponding author:

Diana Dubrall

Institute for Medical Biometry, Informatics and Epidemiology, University Hospital Bonn, Venusberg-Campus 1, 53127 Bonn, Germany

Federal Institute for Drugs and Medical Devices (BfArM), Bonn, Germany

Kurt-Georg-Kiesinger-Allee 3, 53175 Bonn.

Tel: 0228-99-307-5345

E-mail: Diana.Dubrall@bfarm.de

Online Resource 3) DDI-stratified analyses

3.1) Ventricular tachycardia, anticholinergic effects, seizures

	DDI ventricular tachycardia, anticholinergic effects, seizures (n= 40)	pDDI ventricular tachycardia, anticholinergic effects, seizures (n= 234)	P-values
Demographical parameters of the patients			
Mean Age (± SD)	65.8 (±16.6)	57.6 (±19.9)	0.0071*
Female (n, %)	72.5% (n= 29)	59.0% (n= 138)	0.1229
Male (n, %)	27.5% (n= 11)	40.6% (n= 95)	
Unknown (n, %)	0.0% (n= 0)	0.4% (n= 1)	
Seriousness of the ADR reports[1]			
Serious	90.0% (n= 36)	61.5% (n= 144)	0.0002*
Death	5.0% (n= 2)	1.3% (n= 3)	0.1560
Life-threatening	7.5% (n= 3)	5.6% (n= 13)	0.7123
Hospitalisation	57.5% (n= 23)	43.2% (n= 101)	0.1229
Disabling	2.5% (n= 1)	0.0% (n= 0)	-
Primary reporting source[2]			
Physicians	40.0% (n= 16)	43.6% (n= 102)	0.7441
Pharmacists	22.5% (n= 9)	14.1% (n= 33)	0.2254

Other HCP<br><br>Non-HCP	5.0% (n= 2)<br><br>17.5% (n= 7)	6.4 % (n= 15)<br><br>26.1% (n= 61)	1.0000<br><br>0.3253
Most frequently reported histories of the patients with DDI[3]			
NA<br><br>1.<br><br>2.<br><br>3.<br><br>4.<br><br>5.	45.0% (n= 18)<br><br>32.5% Depressed mood disorders and disturbances (n= 13)<br><br>25.0% Vascular hypertensive disorders (n= 10)<br><br>17.5% Schizophrenia and other psychotic disorders (n= 7)<br><br>15.0% Sleep disorders and disturbances (n= 6)<br><br>12.5% Anxiety disorders and symptoms (n= 5)	22.2% (n= 52)<br><br>47.4% Depressed mood disorders and disturbances (n= 111)<br><br>17.9% Vascular hypertensive disorders (n= 42)<br><br>7.7% Schizophrenia and other psychotic disorders (n= 18)<br><br>9.8% Sleep disorders and disturbances (n= 23)<br><br>12.8% Anxiety disorders and symptoms (n= 30)	 <br>0.0830<br><br>0.3763<br><br>0.0590<br><br>0.4078<br><br>1.0000
Number of drugs reported as suspected, interacting or concomitant			
Mean number of drugs (± SD)	7.4 (±4.7)	6.6 (±4.5)	0.2884
Most five most frequently reported drugs as suspected, interacting or concomitant in ADR reports with DDI[4]			
1.<br><br>2.<br><br>3.<br><br>4.<br><br>5.	95.0% Mirtazapine (n= 38)<br><br>40.0% Quetiapine (n= 16)<br><br>30.0% Pantoprazole (n= 12)<br><br>27.5% Risperidone (n= 11)<br><br>22.5% Bisprolol (n= 9)	97.0% Mirtazapine (n= 227)<br><br>30.3% Quetiapine (n= 71)<br><br>17.5% Pantoprazole (n= 41)<br><br>15.4% Risperidone (n= 36)<br><br>14.5% Bisprolol (n= 34)	0.6238<br><br>0.2684<br><br>0.0785<br><br>0.0670<br><br>0.2364

[1] more than one seriousness criterion can be reported in each ADR report. The evaluation of seriousness follows the legal definition. Thus, an ADR report is classified as serious, if the ADR was life-threatening, led to death, disabilities, congenital anomalies or hospitalisation or prolongation thereof.

[2] the primary source qualification describes the person who reported the ADR. More than one primary source qualification can be coded in each ADR report (e.g. physician and consumer reporting about the same ADR). Shown is the number of reports explicitly reported by a physician, pharmacist, consumer or other HCP.

[3] more than one ADR and medical history can be reported in each ADR report. Note that, MedDRA terminology not only describes ADRs but also conditions, laboratory results, diagnoses and investigations.

[4] more than one drug can be reported as suspected/interacting or concomitant in an ADR report. The drugs are classified as suspected, interacting or concomitant by the reporter.

Shown is the descriptive analysis of the ADR reports with and without a DDI of the symptom complex ventricular tachycardia, anticholinergic effects, seizures. For continues variables such as age and the number of drugs reported as suspected, interacting, concomitant Welch two sample t-test was performed. For categorical variables such as sex, primary reporting source, patients' history and reported drugs chi-square test statistic or Fisher's exact test were used whichever was more suitable depending on the sample size.

3.2) Neurotoxic and cardiotoxic effects

	DDI neurotoxic and cardiotoxic effects (n= 29)	pDDI neurotoxic and cardiotoxic effects (n= 71)	P-values
Demographical parameters of the patients			
Mean Age (± SD)	49.9 (±14.6)	49.7 (±16.9)	0.9498
Female (n, %)	37.9% (n= 11)	66.2% (n= 47)	0.0150*
Male (n, %)	62.1% (n= 18)	33.8% (n= 24)	
Unknown (n, %)	0.0% (n= 0)	0.0% (n= 0)	

Seriousness of the ADR reports[1]			
Serious	65.5% (n= 19)	59.2% (n= 42)	0.6567
Death	3.4% (n= 1)	0.0% (n= 0)	-
Life-threatening	13.8% (n= 4)	7.0% (n= 5)	0.4410
Hospitalisation	51.7% (n= 15)	38.0% (n= 27)	0.2624
Disabling	6.7% (n= 2)	0.0% (n= 0)	-
Primary reporting source[2]			
Physicians	58.6% (n= 17)	54.9% (n= 39)	0.8146
Pharmacists	13.8% (n= 4)	5.6% (n= 4)	0.2246
Other HCP	3.4% (n= 1)	7.0% (n= 5)	0.6691
Non-HCP	13.8% (n= 4)	19.7% (n= 14)	0.5767
Most frequently reported histories of the patients with DDI[3]			
NA	10.3% (n= 3)	21.1% (n= 15)	
1.	41.4% Maniac and bipolar mood disorders and disturbances (n= 12)	23.9% Maniac and bipolar mood disorders and disturbances (n= 17)	0.0945
2.	34.5% Schizophrenia and other psychotic disorders (n= 10)	23.9% Schizophrenia and other psychotic disorders (n= 17)	0.3343
3.	24.7% Therapeutic procedures and supportive care (n= 7)	12.7% Therapeutic procedures and supportive care (n= 9)	0.2214
4.	20.7% Depressed mood disorders and disturbances (n= 6)	25.4% Depressed mood disorders and disturbances (n= 18)	0.7871
5.	13.8% Appetite and general nutritional disorders (n= 4)	2.8% Appetite and general nutritional disorders (n= 2)	0.0570

	13.8% Glucose metabolism disorders (incl. diabetes mellitus) (n= 4)	5.6% Glucose metabolism disorders (incl. diabetes mellitus) (n= 4)	0.2246
Number of drugs reported as suspected, interacting or concomitant			
Mean number of drugs (± SD)	5.6 (±2.8)	5.9 (±4.5)	0.6312
Most five most frequently reported drugs as suspected, interacting or concomitant in ADR reports with DDI[4]			
1.	100.0% Lithium (n= 29)	100.0% Lithium (n= 71)	-
2.	41.4% Quetiapine (n= 12)	54.9% Quetiapine (n= 39)	0.2749
3.	34.5% Olanzapine (n= 10)	14.1% Olanzapine (n= 10)	0.0290*
4.	31.0% Aripiprazole (n= 9)	22.5% Aripiprazole (n= 16)	0.4588
5.	31.0% Valproic acid (n= 9)	9.9% Valproic acid (n= 7)	0.0145

[1] more than one seriousness criterion can be reported in each ADR report. The evaluation of seriousness follows the legal definition. Thus, an ADR report is classified as serious, if the ADR was life-threatening, led to death, disabilities, congenital anomalies or hospitalisation or prolongation thereof.

[2] the primary source qualification describes the person who reported the ADR. More than one primary source qualification can be coded in each ADR report (e.g. physician and consumer reporting about the same ADR). Shown is the number of reports explicitly reported by a physician, pharmacist, consumer or other HCP.

[3] more than one ADR and medical history can be reported in each ADR report. Note that, MedDRA terminology not only describes ADRs but also conditions, laboratory results, diagnoses and investigations.

[4] more than one drug can be reported as suspected/interacting or concomitant in an ADR report. The drugs are classified as suspected, interacting or concomitant by the reporter.

Shown is the descriptive analysis of the ADR reports with and without a DDI of the symptom complex neurotoxic and cardiotoxic effects. For continues variables such as age and the number of drugs reported as suspected, interacting, concomitant Welch two sample t-test was performed. For categorical variables such as sex, primary reporting source, patients' history and reported drugs chi-square test statistic or Fisher's exact test were used whichever was more suitable depending on the sample size.

3.3) Ventricular tachycardia, malignant neuroleptic syndrome, serotonin syndrome

	DDI ventricular tachycardia, malignant neuroleptic syndrome, serotonin syndrome (n= 23)	pDDI ventricular tachycardia, malignant neuroleptic syndrome, serotonin syndrome (n= 124)	P-values
Demographical parameters of the patients			
Mean Age (± SD)	51,6 (±15.0)	46.0 (±14.7)	0.1119
Female (n, %)	65.2% (n= 15)	61.3% (n= 76)	0.8016
Male (n, %)	34.8% (n= 8)	38.7% (n= 48)	
Unknown (n, %)	0.0% (n= 0)	0.0% (n= 0)	
Seriousness of the ADR reports[1]			
Serious	56.5% (n= 13)	46.8% (n= 58)	0.4813
Death	8.7% (n= 2)	0.8% (n= 1)	0.0639
Life-threatening	4.3% (n= 1)	4.0% (n= 5)	1.0000
Hospitalisation	43.5% (n= 10)	29.0% (n= 36)	0.2134
Disabling	0.0% (n= 0)	0.8% (n= 1)	-

Primary reporting source[2]			
Physicians	47.8% (n= 11)	33.9% (n= 42)	0.2469
Pharmacists	13.0% (n= 3)	10.5% (n= 13)	0.7176
Other HCP	0.0% (n= 0)	12.9% (n= 16)	-
Non-HCP	26.1% (n= 6)	28.2% (n= 35)	1.0000
Most frequently reported histories of the patients with DDI[3]			
NA	17.4% (n= 4)	23.4% (n= 29)	
1.	60.9% Depressed mood disorders and disturbances (n= 14)	33.9% Depressed mood disorders and disturbances (n= 42)	0.0205*
2.	26.1% Therapeutic procedures and supportive care (n= 6)	16.1% Therapeutic procedures and supportive care (n= 20)	0.3893
3.	17.4% Anxiety disorders and symptoms (n= 4)	16.1% Anxiety disorders and symptoms (n= 20)	1.0000
4.	17.4% Schizophrenia and other psychotic disorders (n= 4)	21.0% Schizophrenia and other psychotic disorders (n= 26)	1.0000
5.	13.0% Mental impairment disorders (n= 3)	2.4% Mental impairment disorders (n= 3)	0.0491*
	13.0% Psychiatric disorders NEC (n= 3)	8.9% Psychiatric disorders NEC (n= 11)	0.4607
	13.0% Psychiatric therapeutic procedures (n= 3)	3.2% Psychiatric therapeutic procedures (n= 4)	0.0771
Number of drugs reported as suspected, interacting or concomitant			
Mean number of drugs (± SD)	5.3 (±2.7)	5.8 (±4.4)	0.5104
Most five most frequently reported drugs as suspected, interacting or concomitant in ADR reports with DDI[4]			
1.	56.5% Aripiprazole (n= 13)	69.4% Aripiprazole (n= 86)	0.3348
2.	47.8% Sertraline (n= 11)	23.4% Sertraline (n= 29)	0.0199*

3.	39.1% Quetiapine (n= 9)	24.2% Quetiapine (n= 30)	0.1944
4.	39.1% Venlafaxine (n= 9)	41.9% Venlafaxine (n= 52)	0.8176
5.	34.8% Amitriptyline (n= 8)	18.5% Amitriptyline (n= 23)	0.0840

[1] more than one seriousness criterion can be reported in each ADR report. The evaluation of seriousness follows the legal definition. Thus, an ADR report is classified as serious, if the ADR was life-threatening, led to death, disabilities, congenital anomalies or hospitalisation or prolongation thereof.

[2] the primary source qualification describes the person who reported the ADR. More than one primary source qualification can be coded in each ADR report (e.g. physician and consumer reporting about the same ADR). Shown is the number of reports explicitly reported by a physician, pharmacist, consumer or other HCP.

[3] more than one ADR and medical history can be reported in each ADR report. Note that, MedDRA terminology not only describes ADRs but also conditions, laboratory results, diagnoses and investigations.

[4] more than one drug can be reported as suspected/interacting or concomitant in an ADR report. The drugs are classified as suspected, interacting or concomitant by the reporter.

Shown is the descriptive analysis of the ADR reports with and without a DDI of the symptom complex Ventricular tachycardia, malignant neuroleptic syndrome, serotonin syndrome. For continues variables such as age and the number of drugs reported as suspected, interacting, concomitant Welch two sample t-test was performed. For categorical variables such as sex, primary reporting source, patients’ history and reported drugs chi-square test statistic or Fisher’s exact test were used whichever was more suitable depending on the sample size.

3.4) Ventricular tachycardia

	DDI tachycardia (n= 62)	pDDI tachycardia (n= 466)	P-values
Demographical parameters of the patients			
Mean Age (± SD)<br>Female (n, %)<br>Male (n, %)<br>Unknown (n, %)	64.2 (±16.4)<br>75.0% (n= 15)<br>25.0% (n= 5)<br>0.0% (n= 0)	50.4 (±19.3)<br>62.7% (n= 292)<br>37.3% (n= 174)<br>0.0% (n= 0)	0.0015*<br>0.3461
Seriousness of the ADR reports[1]			
Serious<br>Death<br>Life-threatening<br>Hospitalisation<br>Disabling	95.0% (n= 19)<br>10.0% (n= 2)<br>15.0% (n= 3)<br>55.0% (n= 11)<br>0.0% (n= 0)	65.2% (n= 304)<br>1.1% (n= 5)<br>4.7% (n= 22)<br>40.8% (n= 190)<br>0.9% (n= 4)	0.0034*<br>0.0299*<br>0.2407<br>0.2509<br>-
Primary reporting source[2]			
Physicians<br>Pharmacists<br>Other HCP<br>Non-HCP	55.0% (n= 11)<br>20.0% (n= 4)<br>5.0% (n= 1)<br>10.0% (n= 2)	48.1% (n= 224)<br>13.3% (n= 62)<br>4.3% (n= 20)<br>22.1% (n= 103)	0.6457<br>0.3328<br>0.5941<br>0.2715
Most frequently reported histories of the patients with DDI[3]			
NA	15.0% (n= 3)	23.4% (n= 109)	

1.	40.0% Depressed mood disorders and disturbances (n= 8)	41.6% Depressed mood disorders and disturbances (n= 194)	1.0000
2.	35.0% Cardiac arrhythmias (n= 7)	2.6% Cardiac arrhythmias (n= 12)	0.0005*
3.	30.0% Vascular hypertensive disorders (n= 6)	12.9% Vascular hypertensive disorders (n= 60)	0.0445*
4.	25.0% Schizophrenia and other psychotic disorders (n= 5)	17.0% Schizophrenia and other psychotic disorders (n= 79)	0.3639
5.	20.0% Appetite and general nutritional disorders (n= 4)	4.3% Appetite and general nutritional disorders (n= 20)	0.0131*
	20.0% Glucose metabolism disorders (incl. diabetes mellitus) (n= 4)	6.0% Glucose metabolism disorders (incl. diabetes mellitus) (n= 28)	0.0355*
	20.0% Lipid metabolism disorders (n= 4)	4.3% Lipid metabolism disorders (n= 20)	0.0131*
	20.0% Thyroid gland disorders (n= 4)	7.7% Thyroid gland disorders (n= 36)	0.0726
Number of drugs reported as suspected, interacting or concomitant			
Mean number of drugs (± SD)	9.2 (±5.6)	5.6 (±3.8)	0.0095
Most five most frequently reported drugs as suspected, interacting or concomitant in ADR reports with DDI[4]			
1.	50.0% Risperidone (n= 10)	27.0% Risperidone (n= 126)	0.0325*
2.	40.0% Quetiapine (n= 8)	47.4% Quetiapine (n= 221)	0.6682
3.	35.0% Citalopram (n= 7)	13.1% Citalopram (n= 61)	0.0135*
4.	35.0% Mirtazapine (n= 7)	17.0% Mirtazapine (n= 79)	0.0655
5.	30.0% Levothyroxine (n= 6)	12.4% Levothyroxine (n= 58)	0.0325*
	30.0% Ramipril (n= 6)	7.5% Ramipril (n= 35)	0.0025*
	30.0% Torasemide (n= 6)	6.4% Torasemide (n= 30)	0.0015*

[1] more than one seriousness criterion can be reported in each ADR report. The evaluation of seriousness follows the legal definition. Thus, an ADR report is classified as serious, if the ADR was life-threatening, led to death, disabilities, congenital anomalies or hospitalisation or prolongation thereof.

[2] the primary source qualification describes the person who reported the ADR. More than one primary source qualification can be coded in each ADR report (e.g. physician and consumer reporting about the same ADR). Shown is the number of reports explicitly reported by a physician, pharmacist, consumer or other HCP.

[3] more than one ADR and medical history can be reported in each ADR report. Note that, MedDRA terminology not only describes ADRs but also conditions, laboratory results, diagnoses and investigations.

[4] more than one drug can be reported as suspected/interacting or concomitant in an ADR report. The drugs are classified as suspected, interacting or concomitant by the reporter.

Shown is the descriptive analysis of the ADR reports with and without a DDI of the symptom complex ventricular tachycardia. For continues variables such as age and the number of drugs reported as suspected, interacting, concomitant Welch two sample t-test was performed. For categorical variables such as sex, primary reporting source, patients' history and reported drugs chi-square test statistic or Fisher's exact test were used whichever was more suitable depending on the sample size.

3.5) Seizures

	DDI Seizures (n= 18)	pDDI Seizures (n= 100)	P-values
Demographical parameters of the patients			
Mean Age (± SD)<br>Female (n, %)<br>Male (n, %)	42.1 (±18.4)<br>55.6% (n= 10)<br>44.4% (n= 8)	46.9 (±17.2)<br>53.0% (n= 53)<br>47.0% (n= 47)	0.3104<br>1.0000

Unknown (n, %)	0.0% (n= 0)	0.0% (n= 0)	
Seriousness of the ADR reports[1]			
Serious	88.9% (n= 16)	48.0% (n= 48)	0.0015*
Death	0.0% (n= 0)	1.0% (n= 1)	-
Life-threatening	5.6% (n= 1)	0.0% (n= 0)	-
Hospitalisation	38.9% (n= 7)	23.0% (n= 23)	0.2294
Disabling	0.0% (n= 0)	0.0% (n= 0)	-
Primary reporting source[2]			
Physicians	22.2% (n= 4)	32.0% (n= 32)	0.5797
Pharmacists	11.1% (n= 2)	9.0% (n= 9)	0.6744
Other HCP	22.2% (n= 4)	16.0% (n= 16)	0.5046
Non-HCP	33.3% (n= 6)	26.0% (n= 26)	0.5637
Most frequently reported histories of the patients with DDI[3]			
NA	27.8% (n= 5)	23.0% (n= 23)	
1.	33.3% Seizures (incl subtypes) (n= 6)	17.0% Seizures (incl subtypes) (n= 17)	0.1914
2.	16.7% Mental impairment disorders (n= 3)	6.0% Mental impairment disorders (n= 6)	0.1387
3.	16.7% Therapeutic procedures and supportive care (n= 3)	12.0% Therapeutic procedures and supportive care (n= 12)	0.6993
4.	11.1% Depressed mood disorders and disturbances (n= 2)	37.0% Depressed mood disorders and disturbances (n= 37)	0.0326*
5.	11.1% Injuries NEC (n= 2)	3.0% Injuries NEC (n= 3)	0.1663

	11.1% Maniac and bipolar mood disorders and disturbances (n= 2)<br>11.1% Nervous system, skull and spine therapeutic procedures (n= 2)<br>11.1% Neurological disorders congential (n= 2)<br>11.1% Schizophrenia and other psychotic disorders (n= 2)	11.0% Maniac and bipolar mood disorders and disturbances (n= 11)<br>3.0% Nervous system, skull and spine therapeutic procedures (n= 3)<br>8.0% Neurological disorders congential (n= 8)<br>6.0% Schizophrenia and other psychotic disorders (n= 6)	1.0000<br>0.1663<br>0.6487<br>0.3517
Number of drugs reported as suspected, interacting or concomitant			
Mean number of drugs (± SD)	5.4 (±5.0)	5.9 (±3.9)	0.6840
Most five most frequently reported drugs as suspected, interacting or concomitant in ADR reports with DDI[4]			
1.	55.6% Valproic acid (n= 10)	22.0% Valproic acid (n= 22)	0.0050*
2.	44.4% Carbamazepin (n= 8)	23.0% Carbamazepin (n= 23)	0.0715
3.	38.9% Aripiprazole (n= 7)	60.0% Aripiprazole (n= 60)	0.1219
4.	27.8% Lamotrigine (n= 5)	19.0% Lamotrigine (n= 19)	0.5238
5.	27.8% Penobarbital (n= 5)	6.0% Penobarbital (n= 6)	0.0123*

[1] more than one seriousness criterion can be reported in each ADR report. The evaluation of seriousness follows the legal definition. Thus, an ADR report is classified as serious, if the ADR was life-threatening, led to death, disabilities, congenital anomalies or hospitalisation or prolongation thereof.

[2] the primary source qualification describes the person who reported the ADR. More than one primary source qualification can be coded in each ADR report (e.g. physician and consumer reporting about the same ADR). Shown is the number of reports explicitly reported by a physician, pharmacist, consumer or other HCP.

[3] more than one ADR and medical history can be reported in each ADR report. Note that, MedDRA terminology not only describes ADRs but also conditions, laboratory results, diagnoses and investigations.

[4] more than one drug can be reported as suspected/interacting or concomitant in an ADR report. The drugs are classified as suspected, interacting or concomitant by the reporter.

Shown is the descriptive analysis of the ADR reports with and without a DDI of the symptom complex seizures. For continues variables such as age and the number of drugs reported as suspected, interacting, concomitant Welch two sample t-test was performed. For categorical variables such as sex, primary reporting source, patients' history and reported drugs chi-square test statistic or Fisher's exact test were used whichever was more suitable depending on the sample size.

3.6) Increased effects of lamotrigine and skin reactions

	DDI Increased effects of lamotrigine and skin reactions (n= 16)	pDDI Increased effects of lamotrigine and skin reactions (n= 38)	P-values
Demographical parameters of the patients			
Mean Age (± SD)	44.0 (±19.6)	42.1 (±14.5)	0.7235
Female (n, %)	25.0% (n= 4)	42.1% (n= 13)	0.3560
Male (n, %)	75.0% (n= 12)	57.9% (n= 22)	
Unknown (n, %)	0.0% (n= 0)	0.0% (n= 0)	
Seriousness of the ADR reports[1]			
Serious	81.3% (n= 13)	57.9% (n= 22)	0.1276

Death	0.0% (n= 0)	0.0% (n= 0)	-
Life-threatening	6.3% (n= 1)	2.6 % (n= 1)	0.5087
Hospitalisation	43.8% (n= 7)	26.3 % (n= 10)	0.2210
Disabling	0.0% (n= 0)	0.0% (n= 0)	-
Primary reporting source[2]			
Physicians	25.0% (n= 4)	39.5% (n= 15)	0.3652
Pharmacists	12.5% (n= 2)	2.6% (n= 1)	0.2064
Other HCP	12.5% (n= 2)	21.1% (n= 8)	0.7047
Non-HCP	31.3% (n= 5)	26.3% (n= 10)	0.7469
Most frequently reported histories of the patients with DDI[3]			
NA	18.8% (n= 3)	18.8% (n= 3)	
1.	81.3% Seizures (incl subtypes) (n= 13)	63.2% Seizures (incl subtypes) (n= 24)	0.3358
2.	18.8% Neurological disorders (n= 3)	15.8% Neurological disorders (n= 6)	1.0000
3.	12.5% Central nervous system vascular disorders (n= 2)	5.3% Central nervous system vascular disorders (n= 2)	0.5732
4.	12.5% Glucose metabolism disorders (incl. diabetes	2.6% Glucose metabolism disorders (incl. diabetes mellitus)	
5.	mellitus) (n= 2)	(n= 1)	
	12.5% Infections - pathogen unspecified (n= 2)	2.6% Infections - pathogen unspecified (n= 1)	0.2064
	12.5% Therapeutic procedures and supportive care (n= 2)	13.2% Therapeutic procedures and supportive care (n= 5)	0.2064
	12.5% Vascular hypertensive disorders (n= 2)	5.3% Vascular hypertensive disorders (n= 2)	1.0000

			0.5732
Number of drugs reported as suspected, interacting or concomitant			
Mean number of drugs (± SD)	5.7 (±3.1)	7.2 (±5.4)	0.2170
Most five most frequently reported drugs as suspected, interacting or concomitant in ADR reports with DDI[4]			
1.	100.0% Lamotrigine (n= 16)	100.0% Lamotrigine (n= 38)	-
2.	100.0% Valproic acid (n= 16)	100.0% Valproic acid (n= 38)	-
3.	25.0% Levetiracetam (n= 4)	36.8 % Levetiracetam (n= 14)	0.5323
4.	18.8% Levothyroxine (n= 3)	13.2% Levothyroxine (n= 5)	0.6816
5.	18.8% Perampanel (n= 3)	15.8% Perampanel (n= 6)	1.0000

[1] more than one seriousness criterion can be reported in each ADR report. The evaluation of seriousness follows the legal definition. Thus, an ADR report is classified as serious, if the ADR was life-threatening, led to death, disabilities, congenital anomalies or hospitalisation or prolongation thereof.

[2] the primary source qualification describes the person who reported the ADR. More than one primary source qualification can be coded in each ADR report (e.g. physician and consumer reporting about the same ADR). Shown is the number of reports explicitly reported by a physician, pharmacist, consumer or other HCP.

[3] more than one ADR and medical history can be reported in each ADR report. Note that, MedDRA terminology not only describes ADRs but also conditions, laboratory results, diagnoses and investigations.

[4] more than one drug can be reported as suspected/interacting or concomitant in an ADR report. The drugs are classified as suspected, interacting or concomitant by the reporter.

Shown is the descriptive analysis of the ADR reports with and without a DDI of the symptom increased effects of lamotrigine and skin reactions. For continues variables such as age and the number of drugs reported as suspected, interacting, concomitant Welch two sample t-test was performed. For categorical variables such as sex,

primary reporting source, patients’ history and reported drugs chi-square test statistic or Fisher’s exact test were used whichever was more suitable depending on the sample size.
