# Supplementary material for: Analysis of drug-drug interactions between psychiatric drugs in spontaneous adverse drug reaction reports from EudraVigilance
Source: Naunyn Schmiedebergs Arch Pharmacol. 2026 Jan 22;399(7):9607–26. doi: 10.1007/s00210-025-04956-5 (PMC13152985; doi:10.1007/s00210-025-04956-5)
Supplement: Supplementary file 4 — (PDF 149 KB) [file 210_2025_4956_MOESM4_ESM.pdf]

Title: Analysis of drug-drug interactions between psychiatric drugs in spontaneous adverse drug reaction reports from EudraVigilance

Journal name: Naunyn-Schmiedeberg's Archives of Pharmacology

Authors:

Diana Dubrall[1,2], Patrick Christ[1,2], Miriam Böhme[2], Martina Hahn[3,4,5], Matthias Schmid[1], Catharina Scholl[2]

[1] Institute for Medical Biometry, Informatics and Epidemiology, University Hospital Bonn, Venusberg-Campus 1, 53127 Bonn, Germany

[2] Research Division, Federal Institute for Drugs and Medical Devices (BfArM), Kurt-Georg-Kiesinger-Allee 3, 53175 Bonn, Germany

[3]Department of mental health, varisano hospital Frankfurt Hoechst, Gotenstr. 6-8, 65929 Frankfurt, Germany

[4]Department of psychiatry, psychosomatics and psychotherapy at the university hospital Frankfurt, Heinrich-Hoffmann-Str. 10, 60528 Frankfurt, Germany

[5]Department of pharmacology and clinical pharmacy at the Philipps-University Marburg, Karl-von-Frisch-Strasse 2, 35043 Marburg, Germany

Corresponding author:

Diana Dubrall

Institute for Medical Biometry, Informatics and Epidemiology, University Hospital Bonn, Venusberg-Campus 1, 53127 Bonn, Germany

Federal Institute for Drugs and Medical Devices (BfArM), Bonn, Germany

Kurt-Georg-Kiesinger-Allee 3, 53175 Bonn.

Tel: 0228-99-307-5345

E-mail: Diana.Dubrall@bfarm.de

Online Resource 4) Interacting drug pairs and their DDI according to the output of ABDATA interaction analysis.

Interacting drug pair	Output of DDI according to ABDATA	
Lamotrigine - Valproinic acid	Increased effects of Lamotrigine and skin reactions	16
Miratazapine - Quetiapine	Ventricular tachycardia, anticholinergic effects, seizures	14
Lithium - Quetiapine	Neurotoxic and cardiotoxic effects	12
Miratazapine - Risperidone	Ventricular tachycardia, anticholinergic effects, seizures	11
Lithium - Olanzapine	Neurotoxic and cardiotoxic effects	10
Aripiprazole - Lithium	Neurotoxic and cardiotoxic effects	9
Lorazepam - Olanzapine	Sedation, cardiorespiratory depression, death	9
Lorazepam - Valproinic acid	Increased effects of lorazepam	9
Lithium - Risperidone	Neurotoxic and cardiotoxic effects	8
Miratazapine - Pipamperone	Ventricular tachycardia, anticholinergic effects, seizures	7
Olanzapine - Valproinic acid	Decreased effects of Olanzapine	7
Aripiprazole - Sertraline	Ventricular tachycardia, anticholinergic effects, seizures	6
Carbamazepine - Valproinic acid	Seizures	6

Miratazapine - Venlafaxine	Ventricular tachycardia, anticholinergic effects, seizures	6
Amitriptyline - Sertraline	Ventricular tachycardia, anticholinergic effects, seizures	5
Aripiprazole - Miratazapine	Ventricular tachycardia, anticholinergic effects, seizures	5
Citalopram - Miratazapine	Increased effects of tetracyclic antidepressants	5
Citalopram - Risperidone	Ventricular tachycardia	5
Lithium - Venlafaxine	Serotonin syndrome and QT prolonation	5
Melperone - Miratazapine	Ventricular tachycardia, anticholinergic effects, seizures	5
Phenobarbital - Valproinic acid	Seizures	5
Aripiprazole - Venlafaxine	Ventricular tachycardia, anticholinergic effects, seizures	4
Biperidene - Haloperidol	Anticholinergic effects	4
Biperidene - Risperidone	Anticholinergic effects	4
Clozapine - Lorazepam	Agranulocytosis, collapse and respiratory failure	4
Clozapine - Risperidone	Agranulocytosis, granulocytopenia	4
Duloxetine - Miratazapine	Increased effects of tetracyclic antidepressants	4
Escitalopram - Miratazapine	Increased effects of tetracyclic antidepressants	4

Miratazapine - Olanzapine	Ventricular tachycardia, anticholinergic effects, seizures	4
Amisulpride - Biperidene	Anticholinergic effects	3
Amitriptyline - Duloxetine	Increased effects of tricyclic antidepressants	3
Amitriptyline - Venlafaxine	Ventricular tachycardia, anticholinergic effects, seizures	3
Aripiprazole - Escitalopram	Ventricular tachycardia, anticholinergic effects, seizures	3
Aripiprazole - Fluoxetine	Ventricular tachycardia, anticholinergic effects, seizures	3
Carbamazepine - Lamotrigine	Seizures	3
Citalopram - Quetiapine	Ventricular tachycardia	3
Clozapine - Quetiapine	Agranulocytosis, granulocytopenia	3
Escitalopram - Risperidone	Ventricular tachycardia	3
Escitalopram - Trimipramine	Increased effects of tricyclic antidepressants	3
Risperidone - Venlafaxine	Ventricular tachycardia	3
Amitriptyline - Quetiapine	Ventricular tachycardia, anticholinergic effects, seizures	2
Biperidene - Olanzapine	Anticholinergic effects	2
Biperidene - Quetiapine	Anticholinergic effects	2
Bupropion - Escitalopram	Seizures	2
Bupropion - Miratazapine	Seizures	2

Bupropion - Venlafaxine	Seizures	2
Clozapine - Lithium	Neurotoxic and cardiotoxic effects	2
Clozapine - Valproinic acid	Agranulocytosis, granulocytopenia	2
Diazepam - Olanzapine	Sedation, cardiorespiratory depression, death	2
Escitalopram - Quetiapine	Ventricular tachycardia	2
Haloperidol - Lithium	Neurotoxic and cardiotoxic effects	2
Levodopa - Quetiapine	Decreased efficacy	2
Lithium - Sertraline	Serotonin syndrome and QT prolonation	2
Miratazapine - Sertraline	Ventricular tachycardia, anticholinergic effects, seizures	2
Quetiapine - Sertraline	Ventricular tachycardia	2
Quetiapine - Valproinic acid	Neutropenia, leukopenia	2
Aripiprazole - Bupropion	Seizures	1
Aripiprazole - Citalopram	Ventricular tachycardia, anticholinergic effects, seizures	1
Biperidene - Flupentixol	Anticholinergic effects	1
Bupropion - Quetiapine	Seizures	1
Clozapine - Escitalopram	Ventricular tachycardia	1
Clozapine - Haloperidol	Agranulocytosis, granulocytopenia	1
Clozapine - Paliperidon	Agranulocytosis, granulocytopenia	1
Clozapine - Venlafaxine	Agranulocytosis, granulocytopenia	1
Duloxetine - Risperidone	Increased effects of risperiodone	1
Levetiracetam - Miratazapine	Ventricular tachycardia	1

Melperone - Sertraline	Ventricular tachycardia	1
Olanzapine - Sertraline	Ventricular tachycardia	1
Pipamperone - Sertraline	Ventricular tachycardia	1
Quetiapine - Trazodone	Ventricular tachycardia, anticholinergic effects, seizures	1
Quetiapine - Venlafaxine	Ventricular tachycardia	1
Trimipramine - Venlafaxine	Ventricular tachycardia, anticholinergic effects, seizures	1

Online Resource 4 shows the list of identified interacting drug pairs during the individual case assessment and their output of DDI according to ABDATA. Notably, the output of ABDATA may represent more than one symptom or symptom complexes.
